# Metal–Ligand Interactions in Scandium Complexes with Radiopharmaceutical Applications

**DOI:** 10.1021/acs.inorgchem.3c02211

**Published:** 2023-11-10

**Authors:** Attila Kovács

**Affiliations:** European Commission, Joint Research Centre (JRC), Karlsruhe, Germany

## Abstract

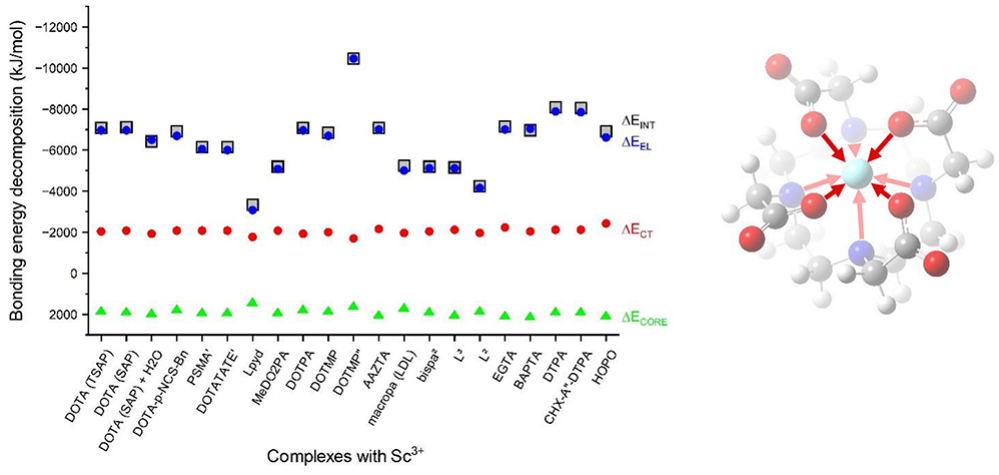

The radioisotopes of scandium (^43^Sc, ^44^Sc, and ^47^Sc) are potential candidates for use in imaging and therapy both separately and as elementally matched pairs for radiotheranostics. In the present study the bonding interactions of Sc^3+^ with 18 hepta- to decadentate ligands are compared using density functional theory (DFT) calculations. The bonding analysis is based on the natural bond orbital (NBO) model. The main contributions to the bonding were assessed using natural energy decomposition analysis (NEDA). Most of the ligands have anionic character (charges from 2– to 8−); thus the electrical term determines the major differences in the interaction energies. However, interesting features were found in the covalent contributions manifested by the ligand → Sc^3+^ charge transfer (CT) interactions. Significant differences could be observed in the energetic contributions of the N and O donors to the total CT.

## Abstract 2

Density functional theory calculations in conjunction with energy decomposition analysis were used to uncover the bonding characteristics of Sc^3+^ complexes. The analysis confirmed the comparable stabilities of complexes with hepta- and octadentate ligands. The primary ligand donors are anionic oxygen and aromatic nitrogen atoms.

## Introduction

Scandium radioisotopes (^43^Sc, ^44^Sc, and ^47^Sc) are important candidates for use in imaging and radioimmunotherapy. Their main advantages are emitted low- or middle-energy β-particles and conveniently short half-lives.

^44^Sc produces middle-energy β^+^ radiation with a high branching ratio (*E*_β^+^_av__ = 632 keV, 94%) and a physical half-life of *t*_1/2_ = 3.97 h.^1^ This large *t*_1/2_ compared with those of the most common β^+^ emitters, ^18^F (1.83 h) and ^68^Ga (1.13 h), facilitates acquiring positron emission tomography (PET) images at later time points than with the latter conventional isotopes. The advantages are a higher image quality,^2^ convenient investigation of pharmacokinetics of slower-circulating bioconjugates, and cost-effective centralized production and regional distribution of the isotope.

^47^Sc is a high branching β^–^ emitter (*E*_β ^–^_av__ = 162 keV, 100%) with a significant half-life (*t*_1/2_ = 3.35 d).^1^ This low-energy radiation is appropriate for treatment of small tumors as well as cancer metastasis,^3^ making ^47^Sc a promising competitor of the clinically established ^177^Lu radionuclide (*E*_β^–^_av__ = 134 keV, *t*_1/2_ = 6.65 d).^1^ In addition, the emitted γ-rays (*E*_γ_ = 159 keV, *I*_γ_ = 68%) have an ideal energy for single photon emission computed tomography (SPECT) imaging.^3^

The above Sc isotopes are perfectly suitable for radiotheranostics, in which method the same molecular targeting vectors are labeled partly with a diagnostic, partly with a therapeutic radionuclide.^4,5^ Currently, the pair of ^68^Ga and ^177^Lu is used in clinics for PET imaging and therapy.^6,7^ However, the two elements have different coordination chemistries^8^ leading to somewhat different in vivo kinetics and receptor binding affinity, thus decreasing the therapeutic efficiency.^9^ From this point of view, the ideal solution would be the combination of different radioisotopes of the same chemical element like the matched pair ^44^Sc and ^47^Sc.

Additional useful Sc radioisotopes include ^43^Sc (*t*_1/2_ = 3.89 h, *E*_β ^+^_av__ = 476 keV, 88%),^1^ which is free from high-energy γ emission and therefore can be suitable for PET imaging.^10^ Last, the ^44m^Sc isotope (*t*_1/2_ = 58.6 h), an isomeric state of ^44^Sc, has been suggested as a potential in vivo ^44^Sc generator allowing longer pharmacokinetic studies.^11^

Currently, the primary chelating ligand for Sc^3+^ is 1,4,7,10-tetraazacyclododecane-1,4,7,10-tetraacetate (DOTA).^12^ Disadvantages of this ligand are, however, its high affinity to other metal ions^13^ and the slow radiolabeling kinetics. There is a high demand for more suitable ligands which could increase the efficiency of Sc radiotheranostics.

In the present work the structural and bonding properties of Sc^3+^ with various cyclic and acyclic chelating ligands are studied in the isolated molecules using DFT calculations. The small ionic radius of Sc^3+^ may imply preferred interaction with small ligands with coordination numbers (CNs) of <8. However, a comparison of available stability constants of Sc^3+^ with octadentate and smaller hexadentate ligands proved the preference for octacoordination.^14,15^ On the other hand, heptacoordination can be reasonably stable.^16^ Therefore, in the present study the bonding characteristics with selected potentially octa- or heptadentate ligands are assessed. They contain various donor groups with the main donor atoms including neutral N and O and anionic (mostly carboxylate) O. The metal–ligand interactions are analyzed using the natural bond orbital (NBO) model.^17−19^

## Ligands

**Figure 1:**
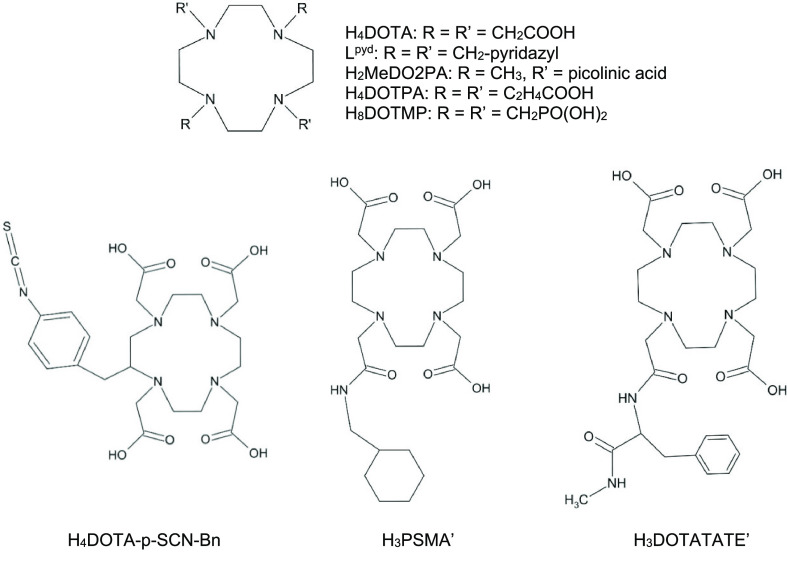
Cyclen-based ligands probed with Sc^3+^ in the present study. H_3_PSMA′ and H_3_DOTATATE′ are truncated models of the very large original ligands.

The cyclen-based ligands presented in Figure 1 compose an important group of chelators in radiopharmaceutics. The 12-membered ring of cyclen (1,4,7,10-tetraazacyclododecane) forms a semirigid basis of the ligand cavity with (generally four) pendant arms filling the conformational space around the captured metal ions. The cyclen ring itself can establish tetradentate coordination with its four N donor atoms.

The most known ligand from the cyclen-based group is H_4_DOTA (1,4,7,10-tetraazacyclododecane-1,4,7,10-tetraacetic acid). Due to its good performance for metal ions, it achieved the title of “gold standard” for a number of trivalent radioisotopes, including ^111^In, ^177^Lu, ^86/90^Y, ^213^Bi, ^225^Ac, and ^44/47^Sc.^12^ The crystal structure of the Sc complex as well as the thermodynamic stability in solution (log *K* = 30.79) was reported by Pniok et al.^20^ The ligand is generally applied in alkaline aqueous solutions; thus the complexes formed between DOTA^4–^ and trivalent metal ions have a charge of 1–. In the complexes generally octacoordination is established through the cyclen N and carboxylate O donors. However, due to the small size of the pendant arms the metals are not fully surrounded, thus allowing coordination of an additional small ligand at the partly open side between the COO^–^ groups of the complex. In aqueous solutions this is generally a H_2_O molecule;^21,22^ its role in complexation of the Sc^3+^ ion is probed here too.

Three extended H_4_DOTA derivatives are included in the present study in order to estimate the effect of popular linker moieties (which link the complex with the targeting vector) on the bonding properties. These linkers are substituents in the outer sphere of the tetraazacyclododecane ring in order to avoid direct interference with metal binding. We probed the *p*-SCN-Bn (4-isothiocyanatophenyl) methyl substituent^23^ in position 2 as well as truncated H_3_PSMA and H_3_DOTATATE ligands, in which one COOH group of H_4_DOTA is replaced by (CO)NH–CH_2_–C_6_H_11_ and (CO)NH–CH(−CH_2_–C_6_H_5_)–(CO)NH–CH_3_ groups, respectively (cf. Figure 1). Note that the kinetic and thermodynamic stabilities of these latter complexes were found to be slightly lower than those of the H_4_DOTA ones, but still sufficiently high for in vivo application.

Neutral ligands are represented in this study by 1,4,7,10-tetrakis(3-pyridazylmethyl)-1,4,7,10-tetraazacyclododecane (L^pyd^). The N donors of the pendant arms facilitate octadentate coordination to the metal ion, whereas the large size of the arms can shield the metal and sterically hinder a coordination of additional small molecules (e.g., H_2_O). This ligand was shown recently to be effective for the borderline Lewis acid Bi^3+^,^24^ and it would be of interest to see how the bonding occurs in detail with the harder Lewis acid Sc^3+^.

The H_2_MeDO2PA (6,6′-((4,10-dimethyl-1,4,7,10-tetraazacyclododecane-1,7-diyl) bis(methylene))dipicolinic acid) ligand has only two pendant arms, but it achieves octacoordination by the pyridine N and carboxylate O donors of the picolinic acid arms. This ligand was found to be superior to H_4_DOTA in complexing Bi^3+^;^25,26^ thus it would be interesting to see the relation in the case of Sc^3+^.

The H_4_DOTPA (1,4,7,10-tetraazacyclododecane-1,4,7,10-tetrapropionic acid) molecule contains pendant arms elongated by a methylene group, and in this way it has a larger flexibility with respect to H_4_DOTA. This larger flexibility seemed to be a slight advantage for bonding with the Bi^3+^ and Ac^3+^ ions as compared to H_4_DOTA.^23^

In the H_8_DOTMP ligand (((1,4,7,10-tetraazacyclododecane-1,4,7,10-tetrayl)tetrakis(methylene))tetraphosphonic acid) the carboxylate oxygen donors of H_4_DOTA are replaced by phosphonate ones. This chelator has been considered for a bone-seeking agent with various radioisotopes (^111^In, ^166^Ho, ^153^Sm, ^177^Lu, ^212^Bi).^27−29^ In this study both the form with the fully deprotonated (charge 8−) and that with the half-deprotonated (charge 4−) tetraphosphonic acid were probed. The two forms facilitate a comparison of the coordination interactions in strong and moderately alkaline environments.

**Figure 2:**
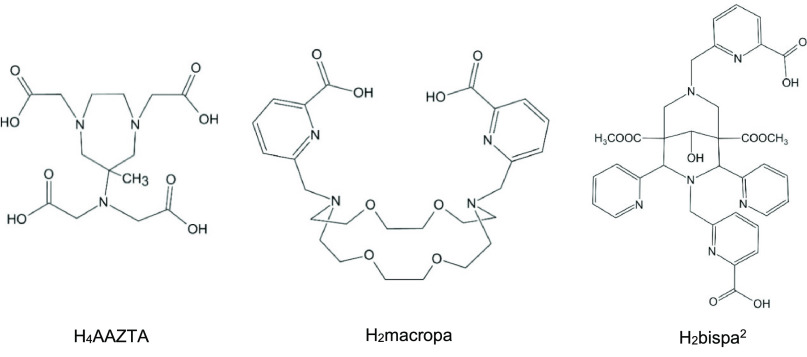
Other applied cyclic ligands. From H_2_bispa^2^ two heptadentate ligands (L^2^, L^3^) were derived by modification of the top picolinic acid moiety; see text.

Figure 2 presents some additional cyclic ligands probed in the present study. From them H_4_AAZTA (1,4-bis(carboxymethyl)-6-(bis(carboxymethyl))-amino-6-methylperhydro-1,4-diazepine) already has a history with Sc^3+^, forming highly stable complexes (log *K* = 27.7).^16^ This heptadentate ligand coordinates by four carboxylate O and three tertiary amine N donors. The backbone is flexible enough for room-temperature radiolabeling, while the semirigid cyclic moiety results in inertness comparable to DOTA.

H_2_macropa (*N*,*N*′-bis((6-carboxy-2-pyridil)methyl)-4,13-diaza-18-crown-6) is a recently introduced competitor for H_4_DOTA for actinides and lanthanides.^30−32^ This decadentate ligand can efficiently capture metal ions with the N and O donors in the crown and picolinate arms. Though H_2_macropa was reported to prefer larger-size metals,^30^ a comparative bonding analysis of its complex with Sc^3+^ can be of interest.

Bispidine-based ligands showed high complex stability, inertness, and fast complexation kinetics^33,34^ and can simply be conjugated to peptides and antibodies.^35−37^ Another advantage of this ligand family is the easy variation of O- and/or N-donor sets on the rigid bispidine scaffold producing CN = 4–8.^33,34,38,39^ The recently introduced octadentate H_2_bispa^2^ (6,6′-((9- hydroxy-1,5-bis(methoxycarbonyl)-2,4-di(pyridin-2-yl)-3,7-diazabicyclo[3.3.1]nonane-3,7-diyl) bis(methylene))dipicolinic acid) provided excellent performances with medically relevant metal ions In^3+^, Bi^3+^, Lu^3+^, and La^3+^ including fast complex formation under ambient conditions.^33,40^ Two other picolinate-/acetate-based heptadentate bispa-type ligands^40^ are also included in the present study in order to check whether they might be more suitable for the small Sc^3+^ ion. They are derived from H_2_bispa^2^ by removing the COOH group from the top picolinic acid (see Figure 2) and by replacing this top picolinic acid with a COOH group, denoted as HL^2^ and H_2_L^3^, respectively.

**Figure 3:**
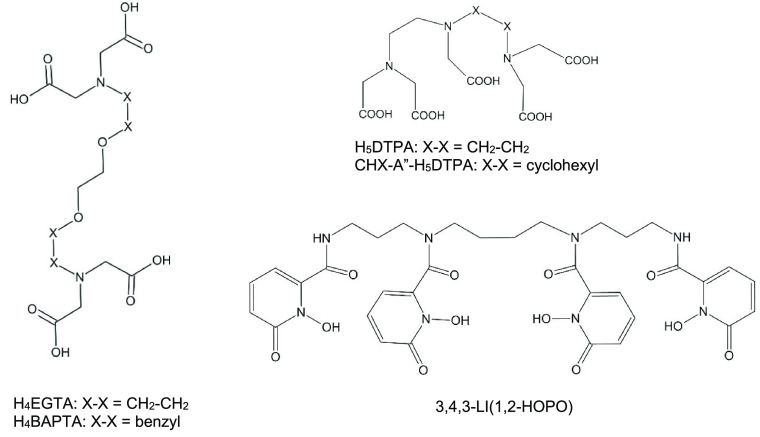
Acyclic ligands probed with Sc^3+^ in the present study.

Acyclic chelators are kinetically somewhat less stable than macrocyclic ones (like H_4_DOTA), but they have the advantage that the formation of their complexes at room temperature is much faster. In the present study five octadentate acyclic chelators (Figure 3) were probed. Three of them have been reported for fast complex formation with Sc^3+^ and very high labeling yields.^41^

H_4_EGTA (ethylene glycol-bis(2-aminoethyl ether)-*N*,*N*,*N*′,*N*′-tetraacetic acid, also known as egtazic acid) showed very good complexation properties to Sc^3+^, outperforming several acyclic ligands, and thus was suggested to be a suitable precursor for Sc radiopharmaceuticals.^41^ The strong complexes are formed by binding via the four carboxylic and two ether O atoms, completing the coordination by two tertiary amine N’s.

H_4_BAPTA (6-dentate 1,2-bis(*o*-aminophenoxy)ethane-*N*,*N*,*N*′,*N*′-tetraacetic acid) is compatible with H_4_EGTA for the binding of Ca^2+^ ions^42^ but for Sc^3+^ showed a worse performance than its parent H_4_EGTA.^41^ Surely, the structure of H_4_BAPTA is less flexible due to substitution of two ethylene moieties by benzyl rings. The present bonding analysis can elucidate the details behind the weaker complexation.

H_5_DTPA (1,1,4,7,7-diethylenetriaminepentaacetic acid) belongs to the most efficient acyclic chelators^12^ due primarily to the five carboxylate O donors and efficient encapsulation of metal ions. The coordination sphere is completed by three tertiary amine N’s (cf. Figure 3). This ligand has been found to exert great affinity for Sc^3+^ (log *K* = 27.43)^20^ and was suggested to be a suitable precursor for Sc radiopharmaceuticals.^41^ The observed dissociation aptitude in solution was alleviated in its more preorganized (semirigid) derivative, CHX-A″-H_5_DTPA (2-aminoethyl-*trans*-[*S*,*S*]-cyclohexane-1,2-diamine-*N*,*N*,*N*′,*N*″,*N*″-pentaacetic acid) while maintaining rapid labeling kinetics. The latter ligand is now widely used with radioimmunoconjugates.^12,43^

3,4,3-LI(1,2-HOPO), *N*,*N*′-1,4-butanediylbis(*N*-(3-(((1,6-dihydro-1-hydroxy-6-oxo-2-pyridinyl)carbonyl)amino)propyl)-1,6-dihydro-1-hydroxy-6-oxo-2-pyridinecarboxamide (denoted as HOPO below), has demonstrated strong affinity for hard metal 3+ and 4+ ions^44,45^ including Sc^3+^,^46^ with log *K*_ScL_ = 25.16.^47^ Advantages of this ligand include strong bonding with the hard O-donating hydroxypyridinone groups, complex formations at lower pH than H_4_DOTA, and fast binding kinetics.

In the discussion below the abbreviations of the deprotonated ligands are used according to the complexing anionic forms (DOTA, etc.).

## Results and Discussion

### Structural Characteristics

**Figure 4:**
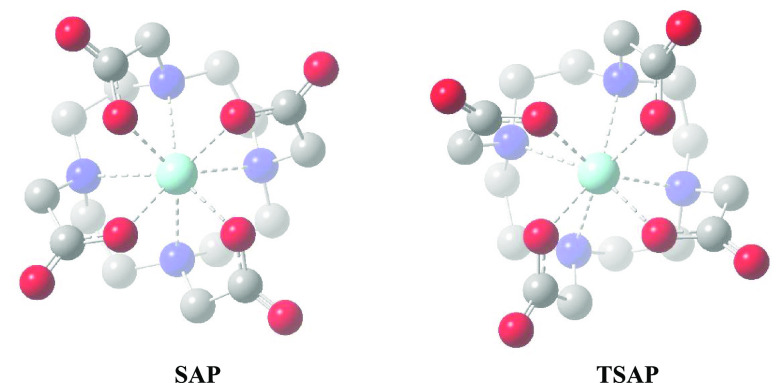
Two conformers of the Sc(DOTA)^−^ complex omitting hydrogen atoms for clarity. Moieties toward the back are gradually faded.

The octacoordinated Sc^3+^ ion has a relatively small effective ionic radius: 0.870 Å.^48^ It is smaller by 0.256 Å than Pr^3+^ (1.126 Å^48^), where the switch of the DOTA conformers in complexes of the lanthanide (Ln) row occurs. Trivalent ions with ionic radii larger than that of Pr^3+^ are known to prefer the twisted square antiprismatic (TSAP) conformer of DOTA, whereas the smaller lanthanides favor the square antiprismatic (SAP) one.^8,49−51^ These two conformers are presented in Figure 4. The SAP structure has slightly smaller cavity and thus can form stronger interactions with the smaller ions. It should be noted that in the crystal of K[Sc(DOTA)](H_6_DOTA)Cl_2_·4H_2_O the TSAP conformer has been identified, which may be stabilized by the interaction of the four COO^–^ groups with K^+^ linking the H_6_(DOTA)Cl_2_ moiety to Sc(DOTA)^−^.^20^

In agreement with expectations, in the present B3LYP/6-31G** calculations the SAP conformer of ScDOTA proved to be more stable by 15.2 kJ/mol than the TSAP conformer. A similar stability relation was computed for the DOTA-*p*-NCS-Bn, DOTATATE′, and PSMA′ ligands. In contrast, the other cyclen-based ligands with larger pendant arms, DOTPA, DOTMP, and L^pyd^, preferred the TSAP conformer by 15–50 kJ/mol at the B3LYP/6-31G** level.

Another ligand with significant conformational varieties is macropa, from which the Δ(*λδλ*)(*λδλ*) and Δ(*δλδ*)(*δλδ*) conformers, denoted by LDL and DLD, respectively, were probed with Sc^3+^ in the present study. For Ln, the LDL form was found to form the most stable complexes with smaller Ln^3+^ (Lu^3+^),^30^ whereas DLD prefers larger ions (e.g., La^3+^, Ac^3+^).^30,52^ In agreement with the above trend, the Sc(macropa_LDL_)^+^ complex was computed at the B3LYP/6-31G** level to be more stable by 15.9 kJ/mol than the Sc(macropa_DLD_)^+^ conformer. The distortion of the *C*_2_ symmetry to *C*_1_ in the macropa_DLD_ complex is likely the consequence of the lesser ability of this macropa conformer to efficiently encapsulate Sc^3+^.

Some of the probed ligands are known to allow the coordination of an additional small ligand, like H_2_O. This is due to insufficient encapsulation of the metal ion, leaving a site with considerable free space in the coordination sphere of the metal. The optimized structures of the present Sc^3+^ complexes were thoroughly inspected from this point of view. The conclusion of this visual inspection is that small ligands can easily coordinate to the significant free site of the cyclen-type and AAZTA complexes, whereas the other ligands encapsulate Sc^3+^ more tightly, leaving not enough space for attack by a small ligand. The effect of a H_2_O ligand at the ninth coordination site on the bonding properties was assessed in the Sc(DOTA_SAP_)^−^ complex; similar effects can be expected for the other related complexes too.

Table 1 compiles the symmetries of the optimized structures. The well-known *C*_4_ symmetry of DOTA complexes^8,23,49−51,53,54^ is preserved in the derivatives with L^pyd^, DOTPA, and DOTMP. Similarly to literature data on Bi^3+^ complexes, optimized structures with *C*_2_ symmetry were obtained for the MeDO2PA, macropa_LDL_, and BAPTA complexes with Sc^3+^.^23^ In contrast, the *C*_2_ symmetry reported for Ln(macropa_DLD_)^+^ complexes^30,52^ was destroyed with the smaller Sc^3+^ ion.

**Table 1:** Selected Structural Characteristics^a^

complex	CN	symmetry	Sc–O_av_	Sc–N_av_
DOTA_TSAP_	8	*C*_4_	2.099 (4)	2.655 (4)
DOTA_SAP_	8	*C*_4_	2.099 (4)	2.617 (4)
DOTA_SAP_ + H_2_O	9	*C*_2_	2.157 (5)	2.740 (4)
DOTA-*p*-NCS-Bn	8	*C*_1_	2.097 (4)	2.631 (4)
PSMA′	8	*C*_1_	2.122 (4)	2.587 (4)
DOTATATE′	8	*C*_1_	2.122 (4)	2.573 (4)
L^pyd^	8	*C*_4_	–	2.422 (8)
MeDO2PA	8	*C*_2_	2.053 (2)	2.455 (6)
DOTPA	8	*C*_4_	2.069 (4)	2.747 (4)
DOTMP	8	*C*_4_	2.097 (4)	2.661 (4)
DOTMP″	8	*C*_4_	2.059 (4)	3.244 (4)
AAZTA	7	*C*_1_	2.091 (4)	2.468 (3)
macropa_LDL_	10	*C*_2_	2.339 (6)	2.730 (4)
bispa^2^	8	*C*_1_	2.048 (2)	2.483 (6)
L^3^	7	*C*_1_	2.006 (2)	2.382 (5)
L^2^	7	*C*_1_	1.985 (1)	2.347 (6)
EGTA	8	*C*_1_	2.230 (6)	2.474 (2)
BAPTA^b^	8	*C*_2_	2.016 (6)	2.687 (2)
DTPA	8	*C*_1_	2.165 (5)	2.599 (3)
CHX-A″-DTPA	8	*C*_1_	2.161 (5)	2.597 (3)
HOPO	8	*C*_1_	2.231 (8)	–

a The Sc^3+^ complexes are denoted by the ligand names given in the first column. The PSMA′ and DOTATATE′ notations mean the truncated ligands to 73 and 77 atoms, respectively; DOTMP and DOTMP″ mean the DOTMP^4–^ and DOTMP^8–^ ligands, respectively. The average Sc–O and Sc–N bond distances are given in angstroms. The number of coordinating O and N atoms are given in parentheses.

b Two Sc–O distances around 3.4 Å in the Sc(BAPTA)^−^ complex are too large for a significant interaction. They were excluded from the average data in order to reflect the very strong character of the relevant Sc–O interactions.

**Figure 5:**
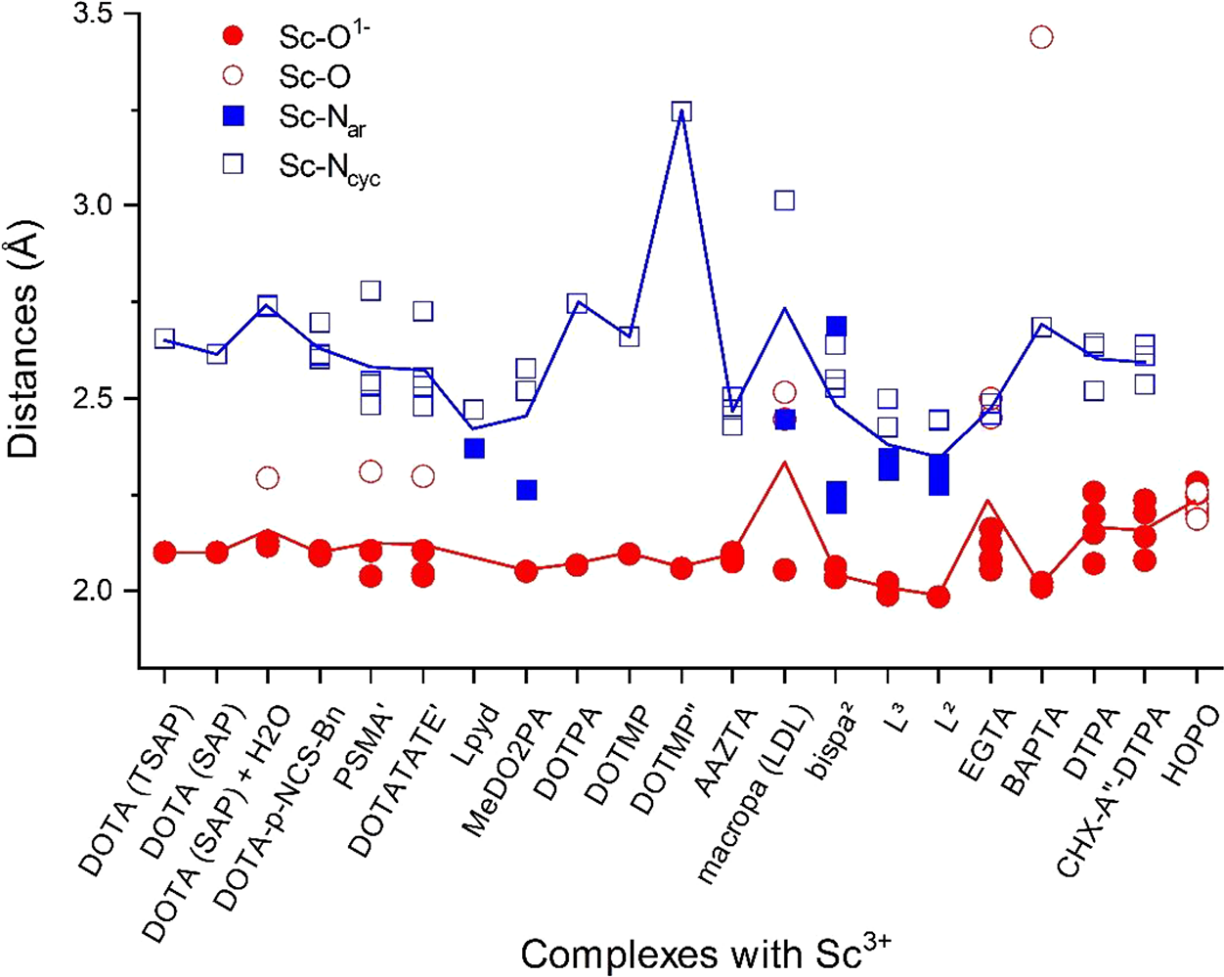
Distributions of Sc–O and Sc–N distances in the studied complexes. For the notation of ligands, see footnote *a* of Table 1. The red and blue lines indicate the average Sc–O and Sc–N values, respectively.

The average Sc–O and Sc–N distances are given in Table 1, whereas the individual bond distances are compiled in Figure 5. The values depicted in Figure 5 as well as the Cartesian coordinates of the optimized structures are given in the Supporting Information. We can distinguish O donors with formal negative charge (carboxylate in most ligands and deprotonated hydroxypyridinone in HOPO, denoted as O^–^) and formally neutral (ether O in macropa, EGTA, BAPTA; C=O in PSMA′, DOTATATE′, and HOPO; denoted as O). The formally neutral N donors can be separated as aliphatic (in cyclen and macropa rings, denoted as N_cyc_) or aromatic (in the picolinate and pyridine/piperidine groups, denoted as N_ar_).

The primary Sc–O distances are around 2.1 Å, whereas those of the Sc–N_ar_ and Sc–N_cyc_ are around 2.3 and 2.6 Å, respectively (cf. Figure 5). Note that the two Sc–O distances around 3.4 Å in the Sc(BAPTA)^−^ complex are too large for a significant interaction; therefore they were excluded from the average data.

The data indicate generally stronger interactions with the O donors than with N, which is reasoned by the anionic character of most O donors in the complexes. The relatively large Sc–O_av_ values in the macropa and EGTA complexes can be attributed to the neutral ether O’s in these ligands, which form very weak interactions with Sc^3+^. The carbonyl oxygens in the HOPO ligand establish intermediate interactions due partly to their better donor abilities with respect to ether O and to their more flexible position in the ligand. Moreover, in HOPO the C=O groups have Sc–O distances comparable to those of the anionic N–O groups implying similar interaction strengths of the two types of O donors.

From the two types of Sc–N interactions, the ones with N_ar_ have generally shorter distances (see Figure 5). As the Sc–N interactions are generally weaker than the Sc–O ones, the latter ones play the major role in the structure of the complexes. Consequently, the bond distances of the weaker Sc–N interactions show a larger variation in Figure 5.

The H_2_O ligand at the ninth coordination site slightly increases the Sc–N_cyc_ distance with respect to that in the parent Sc(DOTA)^−^ complex as it pulls Sc^3+^ slightly closer to the ninth coordination site.

The effects of pendant arms on the Sc–N_cyc_ interaction can be analyzed in the first half of Figure 5 (from DOTA to DOTMP). This effect on the Sc–N_cyc_ bond distances is generally small. A striking exception can be observed in the Sc(DOTMP)^5–^ molecule, where the Sc^3+^ ion is pulled significantly away from the cyclen moiety by the strong electrostatic interactions with the PO_3_^2–^ groups. The Sc–N distance of 3.244 Å suggests marginal interaction with N; thus this complex can rather be referred to as tetracoordinated Sc^3+^. (This marginal Sc–N interaction is reflected also in the CT data, vide infra.) In contrast, the Sc–N distances in the tetraprotonated Sc(DOTMP)^−^ are in the range of those in the complexes with DOTA and other DOTA derivatives. A small but significant increase in the Sc–N_cyc_ distance can be observed also in Sc(DOTPA)^−^, where the pendant arm increased by a CH_2_ group results in an increased cavity affecting primarily the positions of the O donors.

Comparison of the Sc–ligand bond distances in the complexes with DOTA_SAP_ and DOTA_TSAP_ can provide a clue to the stability relations. The Sc–O distances are the same whereas the Sc–N distances are shorter by 0.04 Å in the Sc(DOTA_SAP_)^−^ complex (cf. Table 1 and Figure 5). This refers to comparable Sc–O interactions in the two conformers and indicates that the larger stability of the Sc(DOTA_SAP_)^−^ complex is enforced by its stronger Sc–N interactions. The latter feature is facilitated by the smaller cavity of the SAP structure.

The effect of water solvent on the Sc–donor distances was investigated on selected complexes using the polarizable continuum model (PCM).^55,56^ A figure comparing the average Sc–N and Sc–O distances of the isolated and solvated structures is given in the Supporting Information (Figure S1). The general effect of the water solvent is a slight increase of the Sc–O distances and a minor decrease of the Sc–N ones. The longest Sc–N bonds (with average distances above 2.6 Å) shortened more significantly. The polar solvent changes the polarization of the carboxylate groups and thus reduces the attraction toward Sc^3+^. This gives the possibility to the N donors for a strengthened interaction with Sc^3+^.

### Natural Energy Decomposition Analysis (NEDA)

Briefly, NEDA^57,58^ is an energy partitioning procedure for molecular interactions applicable for self-consistent field (SCF) wave functions and DFT charge densities. The total interaction energy (Δ*E*_int_) between the appropriately selected fragments consists of electrical interaction (EL), charge transfer (CT), and core repulsion (CORE) contributions:
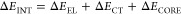


The electrical term, EL = ES + POL + SE, consists of classical electrostatic (ES) and polarization interactions (POL + SE), where SE is the linear response self-energy (energy penalty) of polarization. CT is evaluated as the difference between the energies of the total and localized charge densities. The CORE contribution, CORE = XC + DEF – SE, results from intermolecular exchange–correlation interactions (XC) and deformation (DEF), the latter being the energy difference of the perturbed and relaxed monomer densities.

Metal–DOTA interactions have previously been analyzed in several studies.^8,24,54,59−63^ The main results from the present NEDA study on the isolated complexes are compiled in Table 2. The reported efficient bonding of the DOTA ligand to Sc^3+^ is reflected in the high interaction energy of the Sc(DOTA)^−^ complex in the present set. The other complexes in the present set with 4– charged free ligands have either the same or lower computed interaction energies.

**Table 2:** Selected Results from the NEDA Analysis^a^ of the Isolated Sc Complexes

complex^b^	*q*_c_^c^	Δ*E*_INT_	Δ*E*_EL_	Δ*E*_CT_	Δ*E*_CORE_	%Δ*E*_EL_^d^
DOTA_TSAP_	1–	–7093	–6947	–2033	1887	77.4
DOTA_SAP_	1–	–7109	–6969	–2070	1929	77.1
DOTA_SAP_ + H_2_O^e^	1–	–6440	–6496	–1934	1990	77.1
DOTA-*p*-NCS-Bn	1–	–6936	–6699	–2060	1823	76.5
PSMA′	0	–6159	–6041	–2092	1974	74.3
DOTATATE′	0	–6141	–6017	–2089	1966	74.2
L^pyd^	3+	–3364	–3085	–1753	1474	63.8
MeDO2PA	1+	–5207	–5088	–2081	1963	71.0
DOTPA	1–	–7080	–6974	–1931	1825	78.3
DOTMP	1–	–6839	–6705	–2014	1881	76.9
DOTMP″	5–	–10481	–10452	–1675	1646	86.2
AAZTA	1–	–7084	–6986	–2163	2066	76.4
macropa_LDL_	1+	–5213	–4993	–1950	1729	71.9
bispa^2^	1+	–5211	–5099	–2026	1914	71.6
L^3^	1+	–5154	–5120	–2106	2072	70.9
L^2^	2+	–4238	–4165	–1969	1896	67.9
EGTA	1–	–7149	–7011	–2247	2108	75.7
BAPTA	1–	–6952	–7050	–2051	2149	77.5
DTPA	2–	–8075	–7872	–2110	1907	78.9
CHX-A″-DTPA	2–	–8044	–7844	–2120	1920	78.7
HOPO	1–	–6933	–6628	–2438	2134	73.1

a Energy data (kJ/mol) according to Δ*E*_INT_ = Δ*E*_EL_ + Δ*E*_CT_ + Δ*E*_CORE_. Δ*E*_INT_ means the total interaction energy between the two fragments Sc^3+^ and ligand consisting of electrical interaction (EL), charge transfer (CT), and core repulsion (CORE) contributions.

b The Sc^3+^ complexes are denoted by the ligand names given in the first column. The PSMA′ and DOTATATE′ notations mean the truncated ligands to 73 and 77 atoms, respectively; DOTMP and DOTMP″ mean the DOTMP^4–^ and DOTMP^8–^ ligands, respectively.

c Charges of the isolated complex molecules.

d %Δ*E*_EL_ = 100·Δ*E*_EL_/(Δ*E*_EL_ + Δ*E*_CT_).

e NEDA results of the two-fragment model Sc(H_2_O)^3+^ + DOTA^4–^.

**Figure 6:**
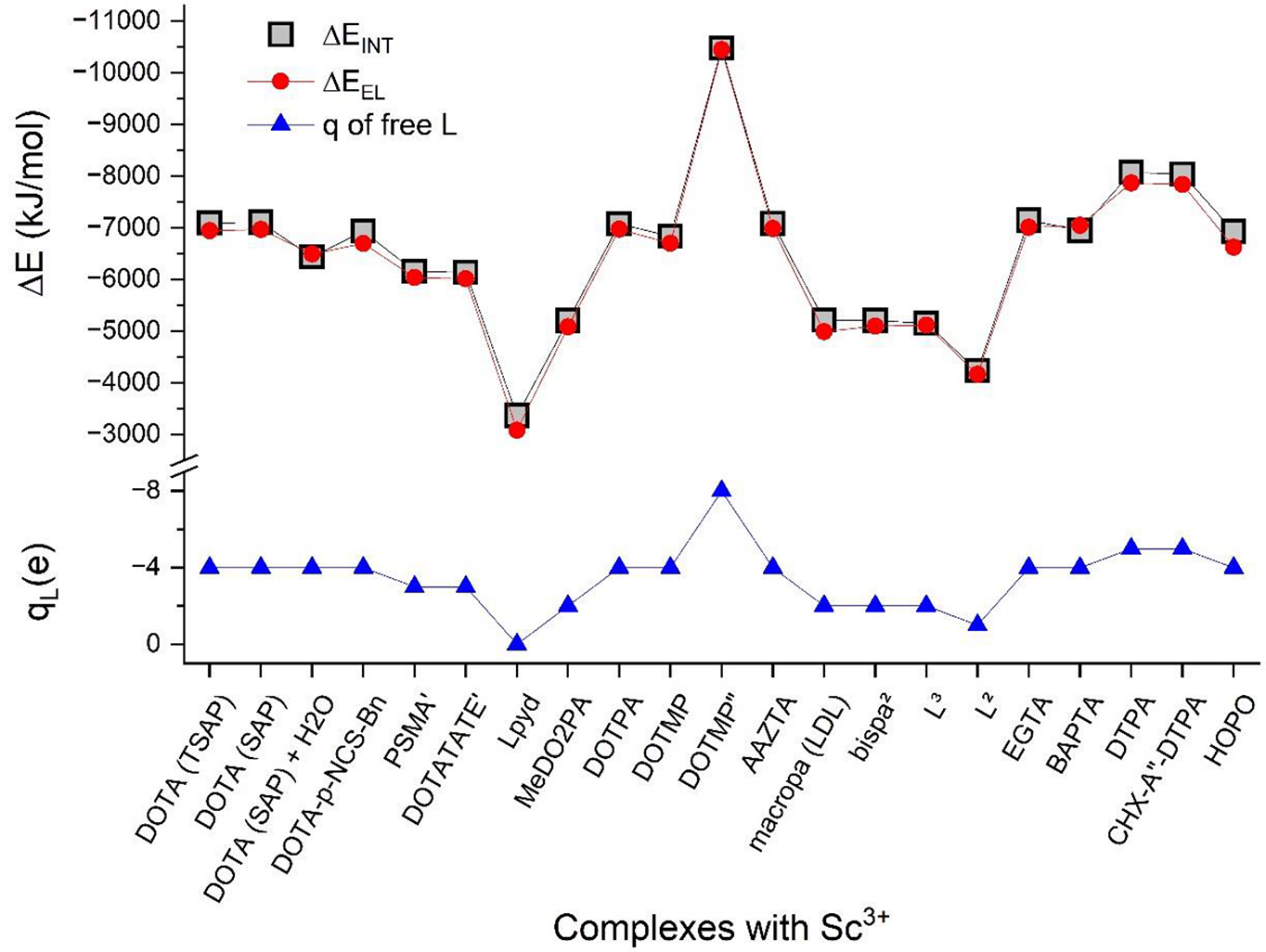
Total interaction energies (Δ*E*_INT_) and electrical contributions (Δ*E*_EL_) of the complexes from the NEDA analysis compared with the formal charges (*q*_L_) of the free ligands. For the notation of ligands, see footnote *a* of Table 1.

The trends in the total interaction energies of the complexes (Δ*E*_INT_), the electrical contributions (Δ*E*_EL_), and formal charges of the free ligands are graphically demonstrated in Figure 6. The total interaction energies between the Sc^3+^ and the ligand fragments vary between −3000 and −11 000 kJ/mol. The trend is primarily determined by the electrical term (EL) due to approximate cancellation of the attractive charge transfer (CT) and repulsive CORE contributions having similar values but opposite signs. The EL contribution is obviously strongly related to the charges of the free ligands: it is the largest with the highly negative DOTMP^8–^ and the smallest with the neutral L^pyd^. This latter significant computed value (−3400 kJ/mol), in spite of the neutral net charge of L^pyd^, originates mainly from the interaction of Sc^3+^ with the negatively polarized N atoms. The roughly linear relation of Δ*E*_INT_ and free ligand charges is well reflected by the gradual increase of Δ*E*_int_ from neutral L^pyd^ < MeDO2PA^2–^, mac^2–^ < DOTA^4–^ derivatives < DTPA^5–^ < DOTMP^8–^ (cf. Figure 6). On the other hand, the markedly different ligand charges do not considerably influence the CT and CORE contributions, as they vary only within 200 kJ/mol (cf. Table 2).

The ratio of the attractive EL and CT interactions is also strongly related to the charges of the free ligands. The dominant (around 70%) EL interactions have a larger ratio in the complexes with highly negative ligands than in those with less negative ones at the cost of CT (cf. Table 2).

Figure 6 provides also a graphical overview on the minor differences of the various derivatives. Attachment of H_2_O, like any additional coordination at the ninth coordination site, decreases slightly the interaction energy of the DOTA complex. In both the two-fragment (Sc(H_2_O)^3+^ + DOTA^4–^) and three-fragment (Sc^3+^ + DOTA^4–^ + H_2_O) models, Δ*E*_INT_ is smaller than the related Sc^3+^ + DOTA^4–^ interaction energy in the Sc(DOTA)^−^ complex. This result is in line with the endothermic dissociation to Sc(DOTA)^−^ + H_2_O from the present B3LYP/6-31G** calculations including a correction for BSSE as well as with literature X-ray diffraction results on K[Sc(DOTA)](H_6_DOTA)Cl_2_·4H_2_O lacking any ninth-coordinated ligand.^20^

The interaction of Sc^3+^ with DOTA is weakened also by the substituents *p*-NCS-Bn, PSMA′, and DOTATATE′. From these latter substituents, the *p*-NCS-Bn one is the most favorable for the complex. It is attached to a cyclen carbon atom and thus leaves all the O and N donors in operation. The slight effect may mainly be attributed to steric interactions. In contrast, the PSMA′ and DOTATATE′ fragments substitute one carboxyl group of DOTA by an amide moiety: accordingly, the strong interaction of Sc^3+^ with the anionic carboxyl O is replaced by a significantly weaker one with a polarized C=O bond of the amide group. As a consequence, Δ*E*_INT_ is decreased by ca. 14%.

The interaction energy of the DOTA complex is preserved upon increasing the pendant arms by CH_2_ groups in Sc(DOTPA)^−^. The lengthening/weakening of the Sc–N bonds noted in the previous section seems to be efficiently compensated by the shortening/strengthening of the Sc–O bonds. In contrast, the slightly longer Sc–N bonds (with preserved Sc–O bond distances) result in a slight decrease of Δ*E*_INT_ for the Sc(DOTMP)^−^ complex.

Essentially the same Δ*E*_INT_ as that with DOTA was computed for the heptadentate AAZTA complex, supporting the experimental observation^16^ and confirming that octacoordination is not a strict requirement for strong metal–ligand interaction in the case of Sc^3+^.

From the acyclic ligands, a Δ*E*_INT_ comparable to that of Sc(DOTA)^−^ is achieved with EGTA, whereas slightly smaller ones are achieved with the BAPTA and HOPO ligands. Thus, the shorter carboxylate O–Sc distances upon benzyl substitution in Sc(BAPTA)^−^ are not enough to increase Δ*E*_INT_ with respect to Sc(EGTA)^−^ as they insufficiently compensate the considerable weakening of the Sc–N and ether O–Sc interactions (see these longer bonds in Figure 5) in the former complex.

There seems to be no gain for the Sc–ligand interaction with introducing a cyclohexyl group in the DTPA^5–^ ligand either. The decreased flexibility of the ligand in CHX-A″-DTPA^5–^ decreased the scattering of the Sc–O and Sc–N distances (cf. Figure 5) and decreased slightly Δ*E*_INT_ with respect to that of Sc(DTPA)^2–^.

The complexes with free ligand charges of 2– (MeDO2PA, macropa, bispa^2^, L^3^) have very close Δ*E*_INT_’s. The marginally smaller value of L^3^ further supports the potential of acyclic heptadentate ligands for complexing Sc^3+^.

### Charge Transfer Interactions

**Figure 7:**
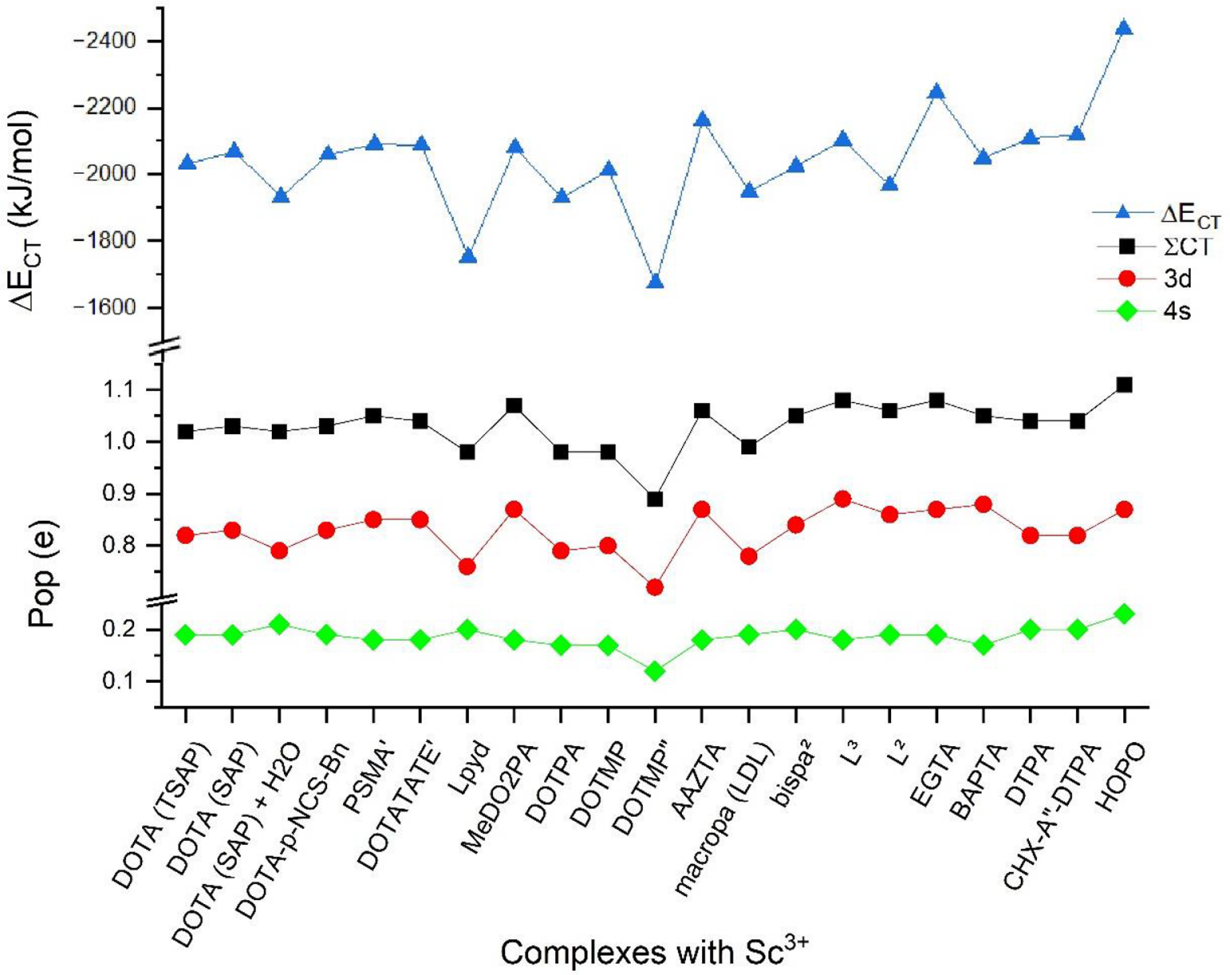
Compilation of the energy contributions of CT interactions in the complexes (Δ*E*_CT_) from the NEDA analyses, the total transferred amount of electrons to Sc^3+^ (∑CT), and natural populations of the acceptor Sc 4s and Sc 3d orbitals from NBO analysis. For the notation of ligands, see footnote *a* of Table 1.

Figure 7 compares the energy contribution of CT interactions from the NEDA analysis with the total transferred amount of electrons to Sc^3+^ and natural populations of the acceptor Sc 4s and Sc 3d orbitals from NBO analysis. There is a reasonably good correlation between the CT energies and transferred electrons. As is known for transition metals, the main electron acceptors are the d orbitals, hence 3d for Sc^3+^, which thus determines the trend for the totally transferred electrons. The population of the 4s orbitals is ca. 25% of those of 3d with small nonsystematic variations.

**Figure 8:**
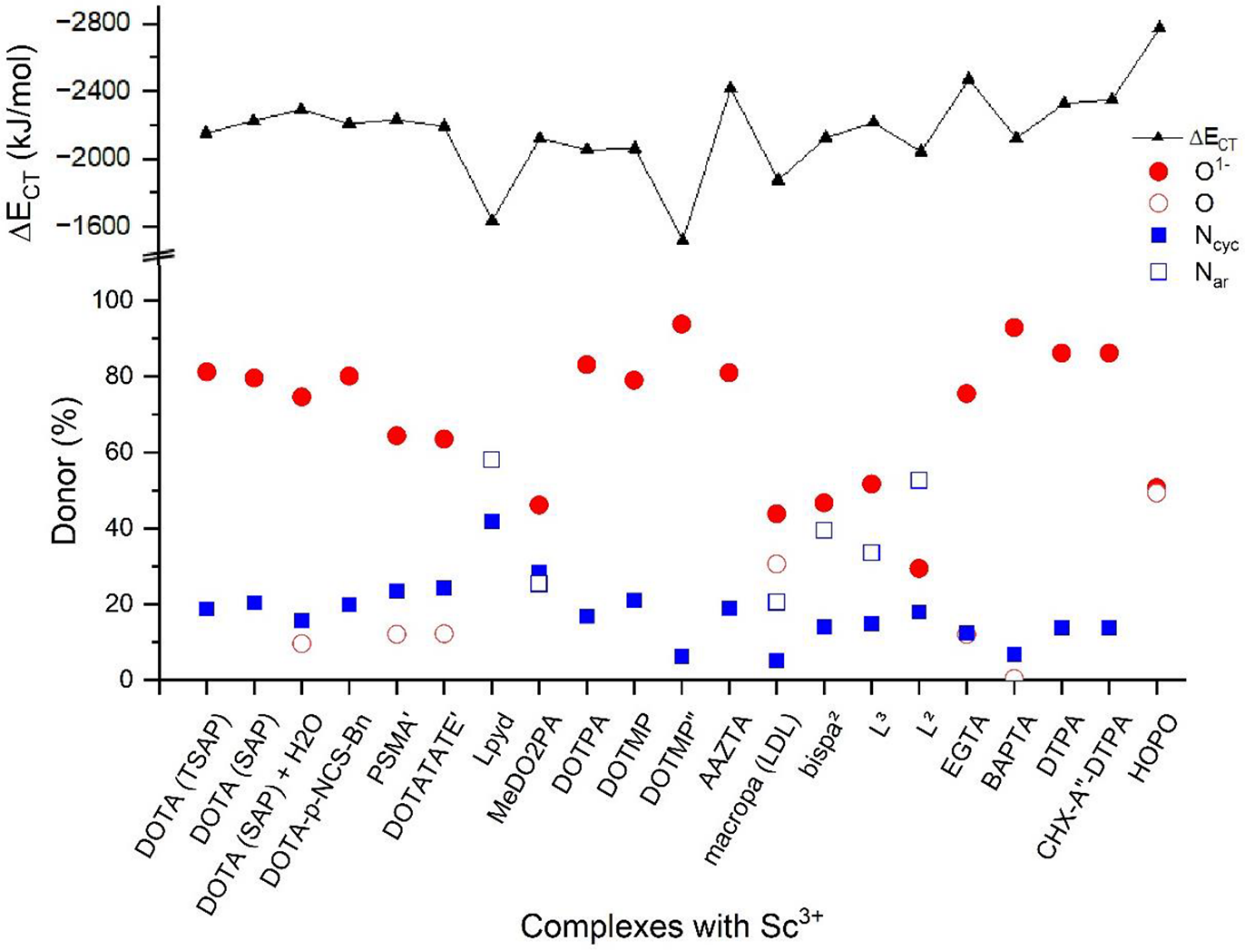
Energetics of CT interactions in the complexes from second-order perturbation theory analysis of the Fock matrix in NBO basis: variation of the total CT energy (Δ*E*_CT_) and % contributions from the four type of donors: anionic O (O^–^), neutral O, aliphatic N (N_cyc_), and aromatic N (N_ar_). The data of Sc(DOTA)(H_2_O)^−^ refer to the three-fragment model: Sc^3+^ + DOTA^4–^ + H_2_O. For the notation of ligands, see footnote *a* of Table 1.

The CT interactions were analyzed in more detail using the second-order perturbation theory approach in the frame of the NBO model. This approach can give the energy contributions of CT between each relevant natural bond orbital and thus facilitates a separation of different types of donors. The contributions of these various donor types in the Sc complexes are summarized in Figure 8.

The curve of the total CT energy from second-order perturbation theory analysis agrees qualitatively with the one from the NEDA analysis (cf. Figures 6 and 7). There are only minor quantitative differences due to the different model theories.

Figure 8 demonstrates the CT predominance of carboxylic O donors in the studied Sc complexes. The lower (%) energetic contribution in the complexes with MeDO2PA, macropa, bispa^2^, L^2^, and L^3^ is in agreement with the small number (one or two) of COO^–^ groups in these ligands. Yet, their cumulative contribution is in most cases higher than that of the (in larger number available) N donors. Interestingly, in the HOPO complex the formally neutral C=O and the formally anionic N–O^–^ donors of the hydroxypyridinone group have nearly identical CT energies. This can be explained by the considerable delocalization of the negative charge in the (O=C—N—O)^−^ moiety (cf. Figure 3) leading to a highly polarized C=O group while at the same time preserving its carbonyl character with a bond distance of 1.26 Å. In contrast, the formally neutral C=O (PSMA′, DOTATATE′) and ring O donors in the other ligands (macropa, EGTA, BAPTA) have low-energy CT contributions.

According to the expectations, the H_2_O ligand in Sc(DOTA)(H_2_O)^−^ has a weak (9.6%) CT to Sc^3+^. Yet, this interaction is considerably stronger than a single Sc–N one (3.95%, summed up to 15.8% of the four N’s). The data in Figure 8 confirm the weakening of both the Sc–O and Sc–N interactions upon H_2_O coordination with respect to the Sc(DOTA)^−^ parent complex.

CT from N donors is generally low (around 20%) except when the ligand contains aromatic N in pyridazine or picoline groups (L^pyd^, macropa, bispa^2^, and derivatives). In the Sc(DOTMP)^5–^ complex the above-mentioned extraordinary move of Sc^3+^ toward the PO_3_^2–^ groups results in marginal N → Sc CT interaction (altogether 6.2% by the four N’s). The similarly weak (6.7%) N → Sc charge donation in the Sc(BAPTA)^−^ complex in spite of the reasonable Sc–N proximity is mainly due to the delocalization of the N lone pairs with the benzene rings in this ligand.

## Conclusions

In the present DFT study the bonding interactions of the pharmaceutically important Sc^3+^ ion with 18 hepta- to decadentate ligands in isolated complex molecules have been assessed. The theoretical analysis was based on natural energy decomposition analysis and second-order perturbation energies of the Fock matrix from the NBO model. The latter results facilitated a differentiation between the various O and N donors for the CT interactions.

The most stable complexes in terms of interaction energies were formed with octa- and heptadentate ligands. With appropriate donors no significant difference was found between the two coordination forms. In contrast, decadentate coordination (with macropa) had no advantage in complex formation. The steric effects originating from the small size of Sc^3+^ and the weak coordination efficiency of ether O donors appeared in several longer Sc–donor distances in this complex.

The computed total interaction energies are differentiated in magnitude of a few thousand kilojoules per mole by the charges of the free ligands from 0 to 8–. In contrast, the covalent charge transfer interactions vary within a few hundred kilojoules per mole only. In agreement with the expectations, in all the complexes (even with the neutral L^pyd^ ligand) the electrical term has the major contribution amounting to 64–86% of the total interaction energy.

More detailed information was obtained on the CT interactions, where both the number of electrons transferred to the acceptor Sc atomic orbitals (3d, 4s) and the energetic contribution of the various O and N donors could be assessed. Δ*E*_CT_ follows qualitatively the trend in the amount of electrons transferred to Sc^3+^, in particular to the 3d orbitals. The 4s orbitals received ca. 20% of the donated electrons and show marginal variations in the complexes.

The energetic analysis of the CT interactions revealed the overwhelming preference of the carboxylate O donors. From N’s, those incorporated in aromatic rings are more efficient donors in spite of their smaller (less negative) natural charges compared to the cyclen N’s.

## Computational Details

The computations were carried out with the Gaussian 09 suite of programs^64^ using the B3LYP exchange–correlation functional^65,66^ in conjunction with the 6-31G** basis set.^67−72^ The B3LYP functional was extended with the D3 version of Grimme’s dispersion correction applying the original D3 damping function.^73^ The SuperFine grid, containing 175 radial shells and 974 angular points (175,974) per shell for H, C, O and N and 250,974 for P, S, and Sc, was applied for integration accuracy. For visualization and construction of some initial geometries, GaussView 5 software^74^ was applied.

The initial structures for geometry optimizations were taken from the following literature data: complexes with DOTA, L^pyd^, MeDO2PA, DOTPA, and DOTMP from computed structures of analogous Bi complexes;^23^ from computed structures of Lu(macropa)^+^,^52^ In(bispa^2^)^+^,^75^ In(L^3^)^+^ and In(L^2^)^2+^;^76^ from the crystal structures of NaGd(AAZTA),^77^ H_2_Bi(DTPA) and H_2_Bi(CHX-A″-DTPA),^78^ KSc(HOPO);^46^ manually generated from the crystal structures of Cd complexes with methoxy-tetrakisquinoline analogues of EGTA and BAPTA.^79^ The applied fragments of the large PSMA and DOTATATE ligands were generated manually starting from the computed Sc(DOTA)^−^ geometries. In addition to geometry optimizations of the isolated complexes, optimizations in aqueous solution using the polarizable continuum model (PCM)^55,56^ were also performed.

The natural atomic charges, valence orbital populations, and second-order perturbation energies were evaluated on the basis of the natural bond orbital model^17^ on the isolated complexes. The metal–ligand interactions were further explored with natural energy decomposition analysis^57,58^ using the NBO 5.9 code^80^ incorporated in Firefly software.^81^ This software is partially based on the GAMESS (US)^82^ source code. The choice of the B3LYP/6-31G** level of theory for this study was primarily determined by the limited availability of exchange–correlation functionals in the Firefly software. A comparison of the computed and available experimental solid-state^20,22^ Sc–O and Sc–N bond distances of Sc(DOTA), Sc(DTPA), and Sc(HOPO) complexes is provided in Table S4 together with a brief discussion in the Supporting Information.

## Supporting Information Available

Average Sc–N and Sc–O distances in isolated and solvated structures of selected complexes; tables with data presented in Figures 4, 6, and 7 and comparing experimental and computed Sc–O and Sc–N bond distances; Cartesian coordinates of relevant optimized structures (PDF)
