# Supplementary material for: Metal–Ligand Interactions in Scandium Complexes with Radiopharmaceutical Applications
Source: Inorg Chem. 2023 Nov 10;62(50):20733–44. doi: 10.1021/acs.inorgchem.3c02211 (PMC10731654; doi:10.1021/acs.inorgchem.3c02211)
Supplement: Supplementary file 1 — ic3c02211_si_001.pdf [file ic3c02211_si_001.pdf]

Supporting Information

Metal-ligand interactions in scandium complexes with radiopharmaceutical applications

Attila Kovács

[a]European Commission, Joint Research Centre (JRC), D-76125 Karlsruhe, Germany

attila.kovacs@ec.europa.eu

Table of Content:

Table S1. Sc-O and Sc-N distances (Å) in the studied complexes (data of Figure 4).
Figure S1. Comparison of the average Sc-O and Sc-N distances in the isolated and solvated structures of selected complexes.
Table S2. ΔE_{CT} (kJ/mol) in the complexes from the NEDA, the total transferred amount of electrons to Sc^{3+} and natural populations of the acceptor Sc 4s and 3d orbitals (e) from NBO analysis (data of Figure 6).
Table S3. CT data from second-order perturbation theory analysis: variation of the total CT energy (ΔE_{CT}) and % contributions from the four type of donors (data of Figure 7).
Table S4. Comparison of experimental and computed Sc-O and Sc-N distances (Å).
Cartesian coordinates of the structures optimized at the B3LYP-D3/6-31G** level

Table S1. Sc-O and Sc-N distances (Å) in the studied complexes.

Complex	Sc-O	Sc-O	Sc-O	Sc-O	Sc-O	Sc-O	Sc-O	Sc-O	Sc-N	Sc-N	Sc-N	Sc-N	Sc-N	Sc-N	Sc-N	Sc-N	$(Sc\text{-}O)_{av}$	$(Sc\text{-}N)_{av}$
$DOTA_{TSAP}$	2.099	2.099	2.099	2.099					2.655	2.655	2.655	2.655					2.099	2.655
$DOTA_{SAP}$	2.099	2.099	2.099	2.099					2.617	2.617	2.617	2.617					2.099	2.617
$DOTA_{SAP}$ + H_2O	2.116	2.129	2.116	2.129	2.294				2.737	2.743	2.737	2.743					2.157	2.740
DOTA-p-NCS-Bn	2.095	2.096	2.103	2.094					2.609	2.603	2.698	2.613					2.097	2.631
PSMA'	2.038	2.037	2.103		2.310				2.546	2.779	2.538	2.483					2.122	2.587
DOTATATE'	2.039	2.046	2.106		2.298				2.531	2.727	2.555	2.48					2.122	2.573
L^{pyd}									2.472	2.472	2.472	2.472	2.372	2.372	2.372	2.372		2.422
MeDO2PA	2.053	2.053							2.520	2.580	2.520	2.580	2.265	2.265			2.053	2.455
DOTPA	2.069	2.069	2.069	2.069					2.747	2.747	2.747	2.747					2.069	2.747
DOTMP	2.097	2.097	2.097	2.097					2.661	2.661	2.661	2.661					2.097	2.661
DOTMP"	2.059	2.059	2.059	2.059					3.244	3.244	3.244	3.244					2.059	3.244
AAZTA	2.077	2.089	2.097	2.100					2.431	2.470	2.503						2.091	2.468
$macropa_{LDL}$	2.054	2.054			2.448	2.448	2.516	2.516	3.013	3.013			2.446	2.446			2.339	2.730
$bispa^2$	2.033	2.063							2.548	2.530	2.638		2.689	2.23	2.262		2.048	2.483
L^3	2.021	1.991							2.499	2.426			2.323	2.313	2.349		2.006	2.382
L^2	1.985								2.441	2.446			2.278	2.329	2.302	2.284	1.985	2.347
EGTA	2.126	2.056	2.085	2.162	2.450	2.499			2.489	2.458							2.230	2.474
BAPTA	2.022	2.022	2.010	2.010	3.437	3.437			2.687	2.687							2.016	2.687
DTPA	2.200	2.257	2.151	2.071	2.144				2.522	2.634	2.642						2.165	2.599
CHX-A"-DTPA	2.078	2.203	2.238	2.141	2.146				2.612	2.538	2.640						2.161	2.597
HOPO	2.282	2.198	2.246	2.211	2.218	2.254	2.186	2.256									2.231	

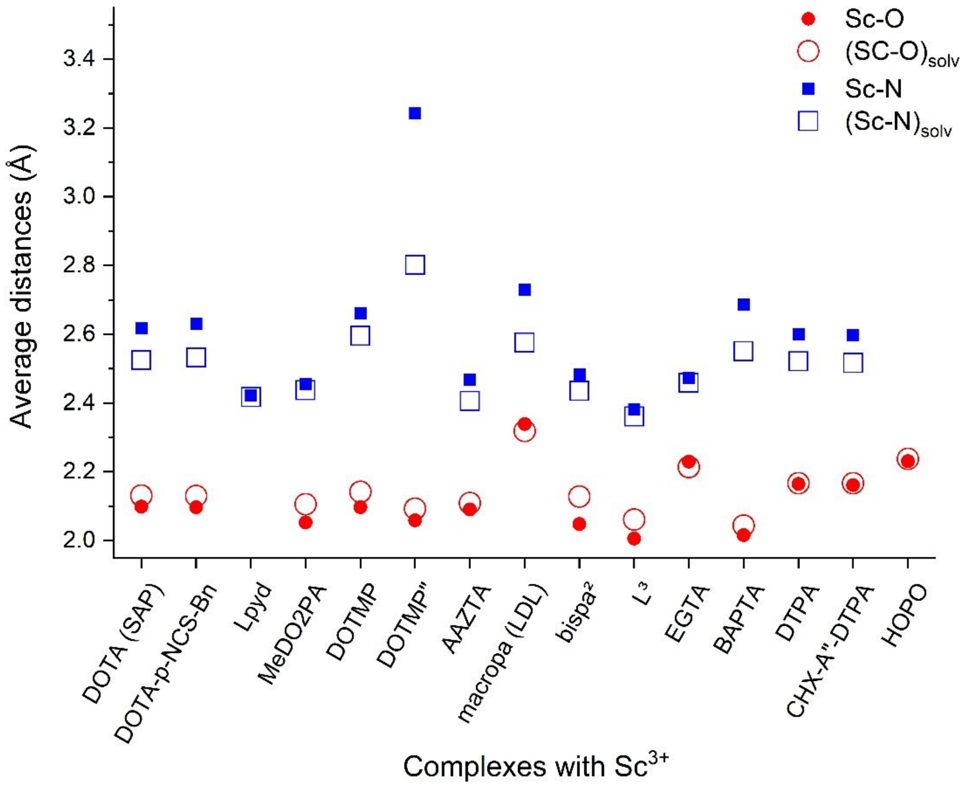

Figure S1. Comparison of the average Sc-O and Sc-N distances in the isolated and solvated structures of selected complexes.

Table S2. ΔE_{CT} (kJ/mol) in the complexes from NEDA, the total transferred amount of electrons to Sc^{3+} and natural populations of the acceptor Sc 4s and 3d orbitals (e) from NBO analysis, natural of Sc (e) in the complexes.

Complex	ΔE_{CT}	e_{CT}	pop 4s	pop 3d	q_{Sc}
$DOTA_{TSAP}$	-2033	1.02	0.19	0.82	1.98
$DOTA_{SAP}$	-2070	1.03	0.19	0.83	1.97
$DOTA_{SAP}$ + H_2O	-2155	1.02	0.21	0.79	1.98
DOTA-p-NCS-Bn	-2060	1.03	0.19	0.83	1.97
PSMA'	-2092	1.05	0.18	0.85	1.95
DOTATATE'	-2090	1.04	0.18	0.85	1.96
L^{pyd}	-1754	0.98	0.20	0.76	2.02
MeDO2PA	-2081	1.07	0.18	0.87	1.93
DOTPA	-1931	0.98	0.17	0.79	2.02
DOTMP	-2014	0.98	0.17	0.80	2.02
DOTMP"	-1675	0.89	0.12	0.72	2.11
AAZTA	-2163	1.06	0.18	0.87	1.94
$macropa_{LDL}$	-1950	0.99	0.19	0.78	2.01
$bispa^2$	-2026	1.05	0.20	0.84	1.95
L^3	-2106	1.08	0.18	0.89	1.92
L^2	-1969	1.06	0.19	0.86	1.94
EGTA	-2247	1.08	0.19	0.87	1.92
BAPTA	-2051	1.05	0.17	0.88	1.95
DTPA	-2110	1.04	0.20	0.82	1.96
CHX-A"-DTPA	-2120	1.04	0.20	0.82	1.96
HOPO	-2438	1.11	0.23	0.87	1.89

Table S3. CT data from second-order perturbation theory analysis: variation of the total CT energy (ΔE_{CT}, kJ/mol) and % contributions from the four type of donors (data of Figure 7).

Complex	ΔE_{CT}	O^-	O	N_{cyc}	N_{ar}
$DOTA_{TSAP}$	-2156	81.2		18.8	
$DOTA_{SAP}$	-2228	79.6		20.4	
$DOTA_{SAP}$ + H_2O	-2295	74.6	9.6	15.8	
DOTA-p-NCS-Bn	-2211	80.1		19.9	
PSMA'	-2235	64.4	12.1	23.4	
DOTATATE'	-2195	63.5	12.2	24.3	
L^{pyd}	-1640			41.9	58.1
MeDO2PA	-2124	46.1		28.5	25.4
DOTPA	-2057	83.1		16.9	
DOTMP	-2064	79		21	
DOTMP"	-1522	93.8		6.2	
AAZTA	-2420	81		19	
$macropa_{LDL}$	-1879	43.8	30.6	5.1	20.5
$bispa^2$	-2129	46.7		14	39.4
L^3	-2218	51.7		14.8	33.5
L^2	-2046	29.4		18	52.6
EGTA	-2475	75.5	12	12.5	
BAPTA	-2126	92.9	0.4	6.7	
DTPA	-2331	86.2		13.8	
CHX-A"-DTPA	-2353	86.2		13.8	
HOPO	-2776	50.7	49.3		

Table S4. Comparison of experimental and computed Sc-O and Sc-N distances (Å).

Crystal	Bond	Experimental	Exp. average	Computed[d]
3(Na[Sc(DOTA)])·NaOH·18H_2O[a]	Sc-O	2.151(2), 2.147(2), 2.163(2)	2.154	2.099
	Sc-N	2.441(3), 2.446(3), 2.450(2)	2.446	2.617
K[Sc(DOTA)][H_6DOTA]Cl_2·4H_2O[b]	Sc-O	2.2228(15)	2.223	2.099
	Sc-N	2.4100(17)	2.410	2.617
[(Hguanidine)]$_2$[Sc(DTPA)]·3H_2O[b]	Sc-O	2.1708(10), 2.1030(9), 2.1883(9), 2.1524(9), 2.2224(9)	2.167	2.165
	Sc-N	2.4496(11), 2.3491(11), 2.4418(11)	2.414	2.559
(K(H_2O)$_2$[Sc(HOPO)])$_2$·2H_2O[c]	Sc-O	2.1790(17), 2.1935(17), 2.1953(19), 2.211(2), 2.2373(17), 2.2385(17), 2.2529(17), 2.2658(17)	2.222	2.231

[a]From Ref. Benetollo et al.: Structural Variations Across the Lanthanide Series of Macrocyclic DOTA Complexes: Insights into the Design of Contrast Agents for Magnetic Resonance Imaging. Inorg. Chem. 2003, 42, 148-157, doi:10.1021/ic025790n.
[b]From Ref. Pniok et al.: Thermodynamic and Kinetic Study of Scandium(III) Complexes of DTPA and DOTA: A Step Toward Scandium Radiopharmaceuticals. Chem. Eur. J. 2014, 20, 7944-7955, doi:10.1002/chem.201402041.
[c]From Ref. Phipps et al.: Sc-HOPO: A Potential Construct for Use in Radioscandium-Based Radiopharmaceuticals. Inorg. Chem. 2023, in press, doi:10.1021/acs.inorgchem.2c03931.
[d]Average bond distances from D3-B3LYP/6-31G** calculations on the $Sc(DOTA)^-$ and $Sc(DTPA)^{2-}$ molecules.

Comments on the data:
1. The Sc-O and Sc-N bond distances differ considerably in the two crystalline compounds of Sc(DOTA) due to the different accompanying compounds and metal ions and arrangements in the crystal structures.
2. The average Sc-O distances computed at the B3LYP-D3/6-31G** level are shorter by several hundredths of Å than the experimental ones in the DOTA complexes, while agree well the ones in the DTPA and HOPO complexes.
3. The average Sc-N distances computed at the B3LYP-D3/6-31G** level are significantly longer than the experimental ones in all complexes. The larger error of the B3LYP exchange-correlation functional for metal-N distances is in agreement with results in Ref. Roca-Sabio et al., Comput. Theor. Chem. 2012, 999, 93–104, doi:10.1016/j.comptc.2012.08.020..

Cartesian coordinates of optimized structures

ScDOTA (TSAP)

Sc	0.000000	0.000000	0.654619
N	0.191155	2.063953	-1.003747
N	-2.063953	0.191155	-1.003747
N	-0.191155	-2.063953	-1.003747
N	2.063953	-0.191155	-1.003747
O	1.097443	1.602346	1.450988
O	-1.602346	1.097443	1.450988
O	-1.097443	-1.602346	1.450988
O	1.602346	-1.097443	1.450988
O	1.305415	3.812039	1.881519
O	-3.812039	1.305415	1.881519
O	-1.305415	-3.812039	1.881519
O	3.812039	-1.305415	1.881519
C	0.880407	2.863391	1.234196
C	-2.863391	0.880407	1.234196
C	-0.880407	-2.863391	1.234196
C	2.863391	-0.880407	1.234196
C	-0.823214	2.074224	-2.063957
C	-2.165771	1.549497	-1.570427
C	-2.074224	-0.823214	-2.063957
C	-1.549497	-2.165771	-1.570427
C	0.823214	-2.074224	-2.063957
C	2.165771	-1.549497	-1.570427
C	2.074224	0.823214	-2.063957
C	1.549497	2.165771	-1.570427
C	0.000000	3.138726	-0.006267
C	-3.138726	0.000000	-0.006267
C	0.000000	-3.138726	-0.006267
C	3.138726	0.000000	-0.006267
H	-0.464219	1.444706	-2.885063
H	-0.959503	3.087217	-2.483005
H	-2.545231	2.210569	-0.790494
H	-2.900184	1.582650	-2.393296
H	-1.444706	-0.464219	-2.885063
H	-3.087217	-0.959503	-2.483005
H	-2.210569	-2.545231	-0.790494
H	-1.582650	-2.900184	-2.393296
H	0.464219	-1.444706	-2.885063
H	0.959503	-3.087217	-2.483005
H	2.545231	-2.210569	-0.790494
H	2.900184	-1.582650	-2.393296
H	1.444706	0.464219	-2.885063
H	3.087217	0.959503	-2.483005
H	2.210569	2.545231	-0.790494
H	1.582650	2.900184	-2.393296
H	0.235231	4.129511	-0.421787
H	-1.033141	3.129876	0.341795
H	-4.129511	0.235231	-0.421787
H	-3.129876	-1.033141	0.341795
H	-0.235231	-4.129511	-0.421787
H	1.033141	-3.129876	0.341795
H	4.129511	-0.235231	-0.421787
H	3.129876	1.033141	0.341795

ScDOTA (SAP)

Sc	0.000000	0.000000	0.706517
N	0.235518	2.073295	-0.873134
N	2.073295	-0.235518	-0.873134
N	-0.235518	-2.073295	-0.873134
N	-2.073295	0.235518	-0.873134
O	-1.131596	1.591479	1.477076
O	1.591479	1.131596	1.477076
O	1.131596	-1.591479	1.477076
O	-1.591479	-1.131596	1.477076
O	3.711059	1.898756	1.452686
O	1.898756	-3.711059	1.452686
O	-3.711059	-1.898756	1.452686
O	-1.898756	3.711059	1.452686
C	-1.119407	2.828015	1.099696
C	2.828015	1.119407	1.099696
C	1.119407	-2.828015	1.099696
C	-2.828015	-1.119407	1.099696
C	1.588902	2.148159	-1.456548
C	2.070568	0.782174	-1.938762
C	2.148159	-1.588902	-1.456548
C	0.782174	-2.070568	-1.938762
C	-1.588902	-2.148159	-1.456548
C	-2.070568	-0.782174	-1.938762
C	-2.148159	1.588902	-1.456548
C	-0.782174	2.070568	-1.938762
C	0.000000	3.170322	0.096364
C	3.170322	0.000000	0.096364
C	0.000000	-3.170322	0.096364
C	-3.170322	0.000000	0.096364
H	0.907643	3.284489	0.691838
H	-0.230472	4.117864	-0.410602
H	4.117864	0.230472	-0.410602
H	3.284489	-0.907643	0.691838
H	0.230472	-4.117864	-0.410602
H	-0.907643	-3.284489	0.691838
H	-3.284489	0.907643	0.691838
H	-4.117864	-0.230472	-0.410602
H	2.273467	2.529646	-0.699408
H	1.623584	2.857556	-2.301092
H	3.077941	0.892585	-2.377419
H	1.417560	0.430063	-2.743191
H	2.857556	-1.623584	-2.301092
H	2.529646	-2.273467	-0.699408
H	0.892585	-3.077941	-2.377419
H	0.430063	-1.417560	-2.743191
H	-1.623584	-2.857556	-2.301092
H	-2.273467	-2.529646	-0.699408
H	-1.417560	-0.430063	-2.743191
H	-3.077941	-0.892585	-2.377419
H	-2.529646	2.273467	-0.699408
H	-2.857556	1.623584	-2.301092
H	-0.430063	1.417560	-2.743191
H	-0.892585	3.077941	-2.377419

ScDOTA (SAP) + H2O

Sc	0.000000	0.000000	0.749572
N	0.227462	2.085672	-1.007263
N	2.090984	-0.246135	-1.008854
N	-0.227462	-2.085672	-1.007263
N	-2.090984	0.246135	-1.008854
O	-1.160647	1.660627	1.360819
O	1.678338	1.198330	1.279326
O	1.160647	-1.660627	1.360819
O	-1.678338	-1.198330	1.279326
O	3.816378	1.908878	1.227730
O	1.857826	-3.801131	1.327017
O	-3.816378	-1.908878	1.227730
O	-1.857826	3.801131	1.327017
C	-1.116383	2.890355	0.960933
C	2.908140	1.145168	0.897751
C	1.116383	-2.890355	0.960933
C	-2.908140	-1.145168	0.897751
C	1.577219	2.142792	-1.596361
C	2.055893	0.771275	-2.071387
C	2.155696	-1.595947	-1.591532
C	0.788427	-2.074750	-2.070378
C	-1.577219	-2.142792	-1.596361
C	-2.055893	-0.771275	-2.071387
C	-2.155696	1.595947	-1.591532
C	-0.788427	2.074750	-2.070378
C	0.000000	3.194424	-0.056556
C	3.209022	-0.011347	-0.073030
C	0.000000	-3.194424	-0.056556
C	-3.209022	0.011347	-0.073030
H	0.909753	3.309994	0.535974
H	-0.224312	4.139574	-0.572529
H	4.155113	0.181590	-0.600494
H	3.312627	-0.905367	0.545719
H	0.224312	-4.139574	-0.572529
H	-0.909753	-3.309994	0.535974
H	-3.312627	0.905367	0.545719
H	-4.155113	-0.181590	-0.600494
H	2.269288	2.535399	-0.852753
H	1.610733	2.838840	-2.453477
H	3.053288	0.888541	-2.533607
H	1.389795	0.413435	-2.862034
H	2.862860	-1.637514	-2.439408
H	2.537764	-2.281894	-0.835448
H	0.900566	-3.079490	-2.517406
H	0.435907	-1.418537	-2.871607
H	-1.610733	-2.838840	-2.453477
H	-2.269288	-2.535399	-0.852753
H	-1.389795	-0.413435	-2.862034
H	-3.053288	-0.888541	-2.533607
H	-2.537764	2.281894	-0.835448
H	-2.862860	1.637514	-2.439408
H	-0.435907	1.418537	-2.871607
H	-0.900566	3.079490	-2.517406
O	0.000000	0.000000	3.043083
H	0.453803	-0.662687	3.571278
H	-0.453803	0.662687	3.571278

ScDOTA-p-NCS-Bn (SAP)

Si	1.965271	-0.033287	-0.858119
N	3.730476	0.692200	0.920640
N	1.081865	1.929063	0.606848
N	-0.232016	-0.737371	0.539369
N	2.472923	-1.977556	0.812817
O	3.843895	-0.777503	-1.411637
O	2.776699	1.763958	-1.567554
O	0.239598	0.689616	-1.817937
O	1.318423	-1.845694	-1.683847
O	3.000212	4.007395	-1.551813
O	-1.981128	1.048684	-1.940331
O	1.071274	-4.086303	-1.683144
O	6.061574	-1.054518	-1.117673
C	4.994517	-0.485529	-0.898125
C	2.424011	2.966109	-1.245361
C	-0.995543	0.425029	-1.543050
C	1.576137	-3.044673	-1.270923
C	3.434207	2.037720	1.448645
C	1.950955	2.202084	1.767148
C	-0.306222	1.701928	1.048864
C	-0.517802	0.277230	1.580670
C	-0.015037	-2.075757	1.120006
C	1.366511	-2.215903	1.757325
C	3.729245	-1.728681	1.547752
C	3.826182	-0.281566	2.022751
C	4.959669	0.695916	0.091013
C	1.151266	3.038061	-0.379227
C	-1.185502	-0.776316	-0.601090
C	2.617651	-3.107600	-0.137784
H	4.938100	1.597334	-0.524283
H	5.867848	0.692896	0.709736
H	1.089994	4.018470	0.113262
H	0.314071	2.921042	-1.069548
H	-2.226918	-0.843357	-0.286720
H	-0.926041	-1.655863	-1.192556
H	3.590663	-3.010170	-0.622483
H	2.555737	-4.078289	0.373563
H	3.730969	2.775851	0.704137
H	4.020942	2.250945	2.358464
H	1.777750	3.221747	2.152774
H	1.673500	1.514928	2.572032
H	-0.585126	2.420674	1.838310
H	-0.967330	1.887745	0.205641
H	0.252416	0.120946	2.344216
H	-0.772824	-2.330717	1.877840
H	-0.124207	-2.813640	0.326209
H	1.462964	-1.503575	2.582524
H	1.447482	-3.222341	2.204276
H	4.568078	-1.953353	0.889524
H	3.823296	-2.398210	2.419494
H	3.015008	-0.076945	2.728274
H	4.769742	-0.149905	2.580864
C	-1.849690	0.141662	2.390312
H	-1.809666	0.928755	3.155707
H	-1.813828	-0.802603	2.942591
C	-3.182337	0.220432	1.676586
C	-4.045006	-0.886876	1.689906
C	-3.607332	1.375347	1.004670
C	-5.276156	-0.860881	1.046446
H	-3.736957	-1.795238	2.200711

C	-4.829067	1.419453	0.344302
H	-2.972612	2.252498	0.977893
C	-5.669794	0.300668	0.364712
H	-5.932440	-1.724741	1.056883
H	-5.134730	2.306835	-0.197727
N	-6.891257	0.351115	-0.280733
C	-7.958578	-0.035308	-0.639769
S	-9.406163	-0.464702	-1.157305

ScPSMA (SAP)

Si	1.198353	0.090769	-0.459196
N	2.115979	1.903653	1.075036
N	0.231972	-0.154477	2.134635
N	1.781794	-2.140649	0.600900
N	3.672711	-0.112755	-0.406976
O	1.720235	1.670357	-1.636609
O	-0.535809	1.524361	0.064371
O	-0.539309	-1.093725	-0.476512
O	1.740948	-1.070955	-2.041657
O	-1.637099	-2.926250	0.209939
O	3.274634	-1.867544	-3.489938
O	2.802024	3.618336	-1.980894
C	2.219487	2.814726	-1.268341
C	-1.456369	0.906049	0.646543
C	-0.605249	-2.332841	-0.102071
C	2.938979	-1.156911	-2.557092
C	1.326794	2.083485	2.314552
C	0.990204	0.765785	2.999524
C	0.389465	-1.551900	2.597334
C	1.679533	-2.172653	2.074395
C	3.158551	-2.499544	0.186764
C	4.110286	-1.314897	0.342460
C	4.299142	1.103609	0.163724
C	3.530723	1.600841	1.386206
C	2.013428	3.120813	0.225689
C	-1.188450	0.214851	1.980127
C	0.765792	-3.028636	-0.029410
C	3.970724	-0.253662	-1.858893
H	0.995040	3.503673	0.312693
H	2.712657	3.901704	0.549187
H	-1.522902	0.889781	2.783258
H	-1.802541	-0.682841	2.042274
H	0.678881	-3.987708	0.496034
H	1.074978	-3.199270	-1.062357
H	3.873854	0.730658	-2.321547
H	4.984258	-0.636125	-2.030062
H	0.398413	2.581378	2.035471
H	1.852136	2.737636	3.028689
H	0.441333	0.979648	3.932522
H	1.910952	0.258439	3.298083
H	0.384476	-1.613781	3.697076
H	-0.457596	-2.140398	2.247121
H	1.747591	-3.208174	2.446828
H	2.538012	-1.634465	2.485832
H	3.543645	-3.346030	0.776552
H	3.133413	-2.822161	-0.852654
H	4.187230	-1.044939	1.399180
H	5.118952	-1.619314	0.024229
H	4.315381	1.878225	-0.603320
H	5.346331	0.917551	0.445521
H	3.552071	0.834070	2.165838
H	4.039388	2.487475	1.797934
N	-2.702480	0.896132	0.166610
H	-2.758422	1.301537	-0.761717
C	-3.731092	-0.104354	0.504553
H	-4.018694	0.017290	1.556126
H	-3.302732	-1.105934	0.365761
C	-4.969274	0.069578	-0.376790
C	-5.954415	-1.088333	-0.135140
C	-5.666673	1.425604	-0.163172

H	-4.640625	0.009159	-1.427148
C	-7.210800	-0.953849	-1.007772
H	-6.248337	-1.087644	0.925190
H	-5.456261	-2.046125	-0.324341
C	-6.921183	1.562620	-1.038243
H	-5.950952	1.506942	0.896694
H	-4.974271	2.252629	-0.361039
C	-7.898660	0.402956	-0.798130
H	-7.905794	-1.773313	-0.791831
H	-6.925616	-1.055223	-2.064141
H	-7.410934	2.523938	-0.844730
H	-6.622552	1.570499	-2.095934
H	-8.768741	0.495325	-1.458365
H	-8.277920	0.459728	0.232279

ScPSMAs (TSAP, 74 atom)

Si	0.311619	-0.001860	-0.634442
N	1.207484	1.777616	1.238397
N	2.293368	-0.918243	0.620410
N	-0.392099	-2.063620	1.085194
N	-1.471320	0.605641	1.667321
C	2.560978	-1.559972	-1.736036
C	2.084133	-2.329378	1.036974
O	3.211591	-2.119236	-2.596679
C	1.186850	3.017460	0.439194
H	1.850994	3.789113	0.853753
H	0.174083	3.420914	0.421119
C	0.458023	1.937371	2.493708
H	0.711974	2.887702	2.992298
H	0.761925	1.142620	3.180513
C	-1.051509	1.878436	2.275862
H	-1.564064	2.049820	3.238389
H	-1.350236	2.685936	1.607617
O	1.841431	3.673955	-1.760435
O	-1.549304	-0.943541	-1.074032
H	2.068967	-2.943266	0.136800
H	2.933971	-2.678348	1.644023
C	3.250061	-0.918061	-0.515429
H	4.173795	-1.460390	-0.275365
H	3.496866	0.110143	-0.785187
C	2.761408	-0.102820	1.758165
H	3.813083	-0.328476	1.996475
H	2.174737	-0.381986	2.638029
C	2.610340	1.390355	1.499817
H	3.025051	1.952934	2.351914
H	3.200604	1.666153	0.625463
O	1.253497	-1.438101	-1.694390
O	1.526377	1.476742	-1.350393
C	1.565761	2.742078	-1.024975
H	0.836179	-2.011413	2.762050
C	-2.737199	0.742364	0.951744
H	-3.476293	1.348566	1.505377
H	-3.184482	-0.238558	0.796664
C	-1.499711	-0.473554	2.661643
H	-2.362519	-0.380350	3.345157
H	-0.605398	-0.374617	3.281873
C	-1.515427	-1.856631	2.024694
H	-1.511287	-2.618569	2.819772
H	-2.451216	-2.007807	1.483646
O	-1.393277	1.854110	-0.716366
H	0.704150	-3.618337	2.060318
C	-2.502322	1.368674	-0.419137
C	-0.848915	-2.948681	-0.012862
H	-1.268966	-3.892881	0.359093
H	-0.014471	-3.150550	-0.687593
C	0.791252	-2.547777	1.808865
C	-1.920750	-2.157553	-0.784867
O	-3.034467	-2.622422	-1.007453
H	-3.308225	1.784124	-2.175212
O	-0.371823	0.470932	-2.886183
H	-1.267718	0.103402	-2.846161
H	-0.441980	1.434582	-2.922349
N	-3.545712	1.412765	-1.264110
C	-4.713739	0.530553	-1.179162
H	-5.391576	0.781127	-1.995932

H	-5.250511	0.692943	-0.240618
H	-4.411108	-0.521265	-1.260856

ScDOTATATE (SAP, 78 atom)

Sc	-1.205833	0.132416	-0.079083
N	-2.138620	-1.352248	-1.904737
N	-0.025158	-2.325252	-0.047796
N	-1.554552	-1.062266	2.152633
N	-3.642766	-0.116675	0.306240
O	-1.945401	1.361748	-1.527902
O	0.428442	-0.152362	-1.668982
O	0.589450	0.205546	1.019300
O	-1.757317	1.616363	1.217641
O	1.843670	-0.227303	2.823840
O	-3.322666	2.895948	2.211686
O	-3.144010	1.736216	-3.399797
C	-2.474192	1.022557	-2.668390
C	1.445392	-0.648750	-1.126885
C	0.752387	-0.119998	2.258533
C	-2.978918	2.026168	1.426849
C	-1.272760	-2.517712	-2.200360
C	-0.781345	-3.221402	-0.941946
C	-0.015191	-2.857329	1.332758
C	-1.298394	-2.514929	2.082761
C	-2.937966	-0.806667	2.613130
C	-3.940079	-0.977974	1.475081
C	-4.314650	-0.653025	-0.902260
C	-3.502798	-1.784426	-1.529761
C	-2.182341	-0.433201	-3.074201
C	1.348979	-2.043793	-0.530612
C	-0.555959	-0.382461	3.016856
C	-4.030970	1.296499	0.571372
H	-1.190286	-0.421961	-3.528432
H	-2.909960	-0.766096	-3.824517
H	1.666971	-2.747792	-1.309209
H	2.066335	-2.162844	0.278351
H	-0.355150	-0.945523	3.937005
H	-0.952694	0.602377	3.271137
H	-4.056686	1.823664	-0.384401
H	-5.016260	1.363843	1.048353
H	-0.410362	-2.146708	-2.753367
H	-1.792561	-3.245296	-2.843530
H	-0.176892	-4.095767	-1.235108
H	-1.633781	-3.612812	-0.380560
H	0.120492	-3.950451	1.340460
H	0.836277	-2.436279	1.866612
H	-1.242903	-2.950536	3.094284
H	-2.150540	-2.986492	1.585460
H	-3.215561	-1.479748	3.439436
H	-2.990000	0.209715	3.001210
H	-3.935653	-2.018238	1.138546
H	-4.953991	-0.775938	1.853173
H	-4.436440	0.157618	-1.620843
H	-5.324849	-1.019369	-0.665613
H	-3.415519	-2.610247	-0.817950
H	-4.044372	-2.176346	-2.405671
N	2.605400	0.016168	-1.145847
H	2.474704	0.946016	-1.535396
C	3.650170	-0.061806	-0.120424
H	3.166367	-0.245298	0.850323
C	4.356949	1.323177	-0.022626
H	5.071275	1.428924	-0.846467
H	4.928564	1.343647	0.912336

C	4.589285	-1.247730	-0.403924
O	4.234644	-2.184956	-1.114167
N	5.790881	-1.210770	0.232678
H	6.001715	-0.427901	0.831388
C	3.361589	2.466258	-0.039096
C	3.264502	3.307047	-1.155938
C	2.434227	2.608626	1.002036
C	2.246337	4.261178	-1.240169
H	3.980416	3.206733	-1.969007
C	1.408113	3.546486	0.910258
H	2.484072	1.941275	1.856063
C	1.309563	4.372530	-0.212778
H	2.177693	4.901665	-2.114422
H	0.660679	3.606061	1.694664
H	0.497728	5.088962	-0.289312
C	6.747179	-2.303772	0.166262
H	6.845618	-2.806011	1.135291
H	6.381653	-3.020572	-0.568970
H	7.730959	-1.938632	-0.144872

ScLpyd (TSAP)

Sc	0.000000	0.000000	-0.435921
N	0.200040	-2.049550	-1.802797
N	-2.049549	-0.200036	-1.802796
N	-0.200040	2.049550	-1.802797
N	2.049549	0.200036	-1.802796
N	1.084546	-1.758519	0.728905
N	-1.758517	-1.084547	0.728902
N	-1.084546	1.758519	0.728905
N	1.758517	1.084547	0.728902
N	-1.718483	1.483293	1.872002
N	1.483291	1.718491	1.871995
N	1.718483	-1.483293	1.872002
N	-1.483291	-1.718491	1.871995
C	-0.819860	-2.073744	-2.890224
H	-0.928938	-3.086352	-3.300345
H	-0.455112	-1.447154	-3.706437
C	-2.163288	-1.568655	-2.400229
H	-2.886846	-1.566698	-3.224932
H	-2.562139	-2.239750	-1.638538
C	-2.073745	0.819860	-2.890226
H	-3.086354	0.928935	-3.300347
H	-1.447155	0.455112	-3.706438
C	-1.568657	2.163289	-2.400235
H	-1.566696	2.886843	-3.224941
H	-2.239753	2.562145	-1.638547
C	0.819860	2.073744	-2.890224
H	0.928938	3.086352	-3.300345
H	0.455112	1.447154	-3.706437
C	2.163288	1.568655	-2.400229
H	2.886846	1.566698	-3.224932
H	2.562139	2.239750	-1.638538
C	2.073745	-0.819860	-2.890226
H	1.447155	-0.455112	-3.706438
H	3.086354	-0.928935	-3.300347
C	1.568657	-2.163289	-2.400235
H	1.566696	-2.886843	-3.224941
H	2.239753	-2.562145	-1.638547
C	0.000000	-3.199874	-0.882524
H	0.270424	-4.144259	-1.373617
H	-1.057528	-3.268593	-0.616317
C	0.784209	-3.020089	0.389656
C	1.135596	-4.106033	1.202313
H	0.903440	-5.121560	0.897319
C	1.776551	-3.830158	2.394040
H	2.075999	-4.615414	3.080375
C	2.044142	-2.481798	2.684575
H	2.549733	-2.187880	3.598807
C	-3.199871	0.000007	-0.882522
H	-4.144258	-0.270411	-1.373614
H	-3.268584	1.057535	-0.616312
C	-3.020087	-0.784207	0.389655
C	-4.106031	-1.135600	1.202310
H	-5.121558	-0.903441	0.897317
C	-3.830156	-1.776564	2.394033
H	-4.615412	-2.076016	3.080365
C	-2.481796	-2.044158	2.684564
H	-2.187877	-2.549756	3.598793
C	0.000000	3.199874	-0.882524
H	-0.270424	4.144259	-1.373617

H	1.057528	3.268593	-0.616317
C	-0.784209	3.020089	0.389656
C	-1.135596	4.106033	1.202313
H	-0.903440	5.121560	0.897319
C	-1.776551	3.830158	2.394040
H	-2.075999	4.615414	3.080375
C	-2.044142	2.481798	2.684575
H	-2.549733	2.187880	3.598807
C	3.199871	-0.000007	-0.882522
H	4.144258	0.270411	-1.373614
H	3.268584	-1.057535	-0.616312
C	3.020087	0.784207	0.389655
C	4.106031	1.135600	1.202310
H	5.121558	0.903441	0.897317
C	3.830156	1.776564	2.394033
H	4.615412	2.076016	3.080365
C	2.481796	2.044158	2.684564
H	2.187877	2.549756	3.598793

ScMeDO2PA

Si	0.000000	0.000000	0.123905
N	-1.126908	1.762743	1.528200
N	1.788019	1.151691	1.584248
N	1.126908	-1.762743	1.528200
N	-1.788019	-1.151691	1.584248
N	0.000000	-2.033059	-0.874174
N	0.000000	2.033059	-0.874174
O	1.624503	0.070858	-1.129992
O	-1.624503	-0.070858	-1.129992
O	2.742736	1.057815	-2.835909
O	-2.742736	-1.057815	-2.835909
C	-0.117066	2.591491	2.247654
H	-0.580564	3.106116	3.100048
H	0.235155	3.369235	1.570354
C	1.062747	1.749324	2.727204
H	1.731220	2.369368	3.344559
H	0.694530	0.947648	3.372307
C	2.741237	0.091166	1.996309
H	3.436967	0.463031	2.764809
H	3.334199	-0.145467	1.110031
C	2.069702	-1.170521	2.511354
H	2.842284	-1.898930	2.796659
H	1.509070	-0.952560	3.423067
C	-1.854340	2.595494	0.525851
H	-2.278810	3.495335	0.992533
H	-2.676214	1.996525	0.123038
C	-0.943288	2.947030	-0.628877
C	-1.059355	4.080557	-1.431666
H	-1.817990	4.827475	-1.223365
C	-0.183211	4.225464	-2.513028
H	-0.258882	5.097335	-3.154953
C	0.786750	3.251891	-2.762164
H	1.490824	3.316936	-3.583327
C	0.842167	2.156481	-1.907354
C	1.844052	1.017827	-2.016353
C	2.592574	2.198981	0.906543
H	1.956107	2.995882	0.529449
H	3.118562	1.759820	0.061088
H	3.328142	2.631947	1.599152
C	0.117066	-2.591491	2.247654
H	0.580564	-3.106116	3.100048
H	-0.235155	-3.369235	1.570354
C	-1.062747	-1.749324	2.727204
H	-1.731220	-2.369368	3.344559
H	-0.694530	-0.947648	3.372307
C	-2.741237	-0.091166	1.996309
H	-3.436967	-0.463031	2.764809
H	-3.334199	0.145467	1.110031
C	-2.069702	1.170521	2.511354
H	-2.842284	1.898930	2.796659
H	-1.509070	0.952560	3.423067
C	1.854340	-2.595494	0.525851
H	2.278810	-3.495335	0.992533
H	2.676214	-1.996525	0.123038
C	0.943288	-2.947030	-0.628877
C	1.059355	-4.080557	-1.431666
H	1.817990	-4.827475	-1.223365
C	0.183211	-4.225464	-2.513028
H	0.258882	-5.097335	-3.154953

C	-0.786750	-3.251891	-2.762164
H	-1.490824	-3.316936	-3.583327
C	-0.842167	-2.156481	-1.907354
C	-1.844052	-1.017827	-2.016353
C	-2.592574	-2.198981	0.906543
H	-1.956107	-2.995882	0.529449
H	-3.118562	-1.759820	0.061088
H	-3.328142	-2.631947	1.599152

ScDOTPA (TSAP)

Sc	0.000000	0.000000	0.633076
N	-0.972485	1.912867	-1.081237
N	-1.912867	-0.972485	-1.081237
N	1.912867	0.972485	-1.081237
N	0.972485	-1.912867	-1.081237
O	1.797645	-0.682960	1.396839
O	-0.682960	-1.797645	1.396839
O	-1.797645	0.682960	1.396839
O	0.682960	1.797645	1.396839
O	-1.613106	-3.828224	1.381933
O	-3.828224	1.613106	1.381933
O	1.613106	3.828224	1.381933
O	3.828224	-1.613106	1.381933
C	-1.698514	-2.595409	1.309417
C	-1.446912	-2.242762	-1.677973
C	-1.923527	3.047186	1.052604
H	-2.680551	3.828965	1.148759
H	-1.152576	3.179092	1.812899
C	0.000000	2.191177	-2.154176
H	-0.239056	3.145809	-2.657445
H	-0.101508	1.416845	-2.918593
C	1.446912	2.242762	-1.677973
H	2.086979	2.521794	-2.535579
H	1.564203	3.034597	-0.939479
H	-1.564203	-3.034597	-0.939479
H	-2.086979	-2.521794	-2.535579
C	-3.047186	-1.923527	1.052604
H	-3.828965	-2.680551	1.148759
H	-3.179092	-1.152576	1.812899
C	-2.191177	0.000000	-2.154176
H	-3.145809	-0.239056	-2.657445
H	-1.416845	-0.101508	-2.918593
C	-2.242762	1.446912	-1.677973
H	-2.521794	2.086979	-2.535579
H	-3.034597	1.564203	-0.939479
C	-2.595409	1.698514	1.309417
H	0.101508	-1.416845	-2.918593
C	3.047186	1.923527	1.052604
H	3.828965	2.680551	1.148759
H	3.179092	1.152576	1.812899
C	2.191177	0.000000	-2.154176
H	3.145809	0.239056	-2.657445
H	1.416845	0.101508	-2.918593
C	2.242762	-1.446912	-1.677973
H	2.521794	-2.086979	-2.535579
H	3.034597	-1.564203	-0.939479
H	0.239056	-3.145809	-2.657445
C	1.698514	2.595409	1.309417
C	1.923527	-3.047186	1.052604
H	2.680551	-3.828965	1.148759
H	1.152576	-3.179092	1.812899
C	0.000000	-2.191177	-2.154176
C	2.595409	-1.698514	1.309417
C	-1.223838	3.178256	-0.324260
H	-1.814601	3.840133	-0.981984
H	-0.263935	3.660587	-0.147419
C	-3.178256	-1.223838	-0.324260
H	-3.840133	-1.814601	-0.981984
H	-3.660587	-0.263935	-0.147419

C	1.223838	-3.178256	-0.324260
H	1.814601	-3.840133	-0.981984
H	0.263935	-3.660587	-0.147419
C	3.178256	1.223838	-0.324260
H	3.840133	1.814601	-0.981984
H	3.660587	0.263935	-0.147419

ScDOTPA (SAP)

Sc	0.000000	0.000000	0.716111
N	0.134384	2.150138	-1.055048
N	2.150138	-0.134384	-1.055048
N	-0.134384	-2.150138	-1.055048
N	-2.150138	0.134384	-1.055048
O	-1.226584	1.490569	1.445923
O	1.490569	1.226584	1.445923
O	1.226584	-1.490569	1.445923
O	-1.490569	-1.226584	1.445923
O	2.743594	3.060819	1.769854
O	3.060819	-2.743594	1.769854
O	-2.743594	-3.060819	1.769854
O	-3.060819	2.743594	1.769854
C	-1.930891	2.558130	1.309520
C	2.558130	1.930891	1.309520
C	1.930891	-2.558130	1.309520
C	-2.558130	-1.930891	1.309520
C	1.478253	2.220959	-1.666672
C	2.043541	0.878005	-2.116806
C	2.220959	-1.478253	-1.666672
C	0.878005	-2.043541	-2.116806
C	-1.478253	-2.220959	-1.666672
C	-2.043541	-0.878005	-2.116806
C	-2.220959	1.478253	-1.666672
C	-0.878005	2.043541	-2.116806
C	0.000000	3.399150	-0.239148
C	3.399150	0.000000	-0.239148
C	0.000000	-3.399150	-0.239148
C	-3.399150	0.000000	-0.239148
H	0.764010	3.344850	0.533765
H	0.239189	4.260656	-0.891063
H	4.260656	-0.239189	-0.891063
H	3.344850	-0.764010	0.533765
H	-0.239189	-4.260656	-0.891063
H	-0.764010	-3.344850	0.533765
H	-3.344850	0.764010	0.533765
H	-4.260656	0.239189	-0.891063
H	2.141458	2.682847	-0.941879
H	1.469424	2.898568	-2.541093
H	3.026658	1.056094	-2.591034
H	1.400219	0.472037	-2.903966
H	2.898568	-1.469424	-2.541093
H	2.682847	-2.141458	-0.941879
H	1.056094	-3.026658	-2.591034
H	0.472037	-1.400219	-2.903966
H	-1.469424	-2.898568	-2.541093
H	-2.141458	-2.682847	-0.941879
H	-1.400219	-0.472037	-2.903966
H	-3.026658	-1.056094	-2.591034
H	-2.682847	2.141458	-0.941879
H	-2.898568	1.469424	-2.541093
H	-0.472037	1.400219	-2.903966
H	-1.056094	3.026658	-2.591034
C	1.330722	-3.682265	0.443789
H	1.167856	-4.538700	1.108885
H	2.104576	-4.012305	-0.258178
C	-3.682265	-1.330722	0.443789
H	-4.538700	-1.167856	1.108885
H	-4.012305	-2.104576	-0.258178

C	-1.330722	3.682265	0.443789
H	-1.167856	4.538700	1.108885
H	-2.104576	4.012305	-0.258178
C	3.682265	1.330722	0.443789
H	4.538700	1.167856	1.108885
H	4.012305	2.104576	-0.258178

ScDOTMP (TSAP)

Sc	0.000000	0.000000	0.186748
N	-2.094002	0.000000	-1.455586
N	0.000000	2.094002	-1.455586
N	2.094002	0.000000	-1.455586
N	0.000000	-2.094002	-1.455586
O	-1.572911	-1.083796	1.052375
O	-1.083796	1.572911	1.052375
O	1.572911	1.083796	1.052375
O	1.083796	-1.572911	1.052375
O	-4.075573	-1.816633	1.031317
O	-1.816633	4.075573	1.031317
O	4.075573	1.816633	1.031317
O	1.816633	-4.075573	1.031317
O	-0.398013	-3.328303	2.182382
O	-3.328303	0.398013	2.182382
O	3.328303	-0.398013	2.182382
O	0.398013	3.328303	2.182382
P	-3.094947	-0.694631	1.014293
P	-0.694631	3.094947	1.014293
P	3.094947	0.694631	1.014293
P	0.694631	-3.094947	1.014293
C	-3.216163	0.321600	-0.541574
H	-4.186989	0.144467	-1.027673
H	-3.151203	1.368166	-0.252108
C	-1.974239	1.007403	-2.523945
H	-2.959057	1.227536	-2.970052
H	-1.358546	0.584573	-3.323523
C	-1.347858	2.303718	-2.028349
H	-1.308588	3.030259	-2.857384
H	-1.975156	2.754031	-1.257331
C	0.321600	3.216163	-0.541574
H	0.144467	4.186989	-1.027673
H	1.368166	3.151203	-0.252108
C	1.007403	1.974239	-2.523945
H	1.227536	2.959057	-2.970052
H	0.584573	1.358546	-3.323523
C	2.303718	1.347858	-2.028349
H	3.030259	1.308588	-2.857384
H	2.754031	1.975156	-1.257331
C	3.216163	-0.321600	-0.541574
H	4.186989	-0.144467	-1.027673
H	3.151203	-1.368166	-0.252108
C	1.974239	-1.007403	-2.523945
H	2.959057	-1.227536	-2.970052
H	1.358546	-0.584573	-3.323523
C	1.347858	-2.303718	-2.028349
H	1.308588	-3.030259	-2.857384
H	1.975156	-2.754031	-1.257331
C	-0.321600	-3.216163	-0.541574
H	-0.144467	-4.186989	-1.027673
H	-1.368166	-3.151203	-0.252108
C	-1.007403	-1.974239	-2.523945
H	-1.227536	-2.959057	-2.970052
H	-0.584573	-1.358546	-3.323523
C	-2.303718	-1.347858	-2.028349
H	-3.030259	-1.308588	-2.857384
H	-2.754031	-1.975156	-1.257331
H	2.573616	-1.026012	2.116959
H	-1.026012	-2.573616	2.116959

H	-2.573616	1.026012	2.116959
H	1.026012	2.573616	2.116959

ScDOTMP" (TSAP)

Sc	0.000000	0.000000	0.986432
N	1.932122	0.981557	-1.427154
N	0.981235	-1.930325	-1.425635
N	-1.932122	-0.981557	-1.427154
N	-0.981235	1.930325	-1.425635
O	1.231228	1.596619	1.402212
O	1.595259	-1.230880	1.407836
O	-1.231228	-1.596619	1.402212
O	-1.595259	1.230880	1.407836
O	2.416154	3.694805	0.433882
O	3.693642	-2.412716	0.435800
O	-2.416154	-3.694805	0.433882
O	-3.693642	2.412716	0.435800
O	-2.118018	3.751755	2.004120
O	3.751598	2.117541	2.003417
O	-3.751598	-2.117541	2.003417
O	2.118018	-3.751755	2.004120
P	2.615774	2.269564	0.994208
P	2.268793	-2.614198	0.996560
P	-2.615774	-2.269564	0.994208
P	-2.268793	2.614198	0.996560
C	3.044836	1.155194	-0.467450
H	3.951067	1.534464	-0.996913
H	3.280872	0.177945	-0.037785
C	2.205544	-0.000303	-2.460194
H	3.183019	0.214040	-2.969908
H	1.441805	0.110860	-3.242526
C	2.241136	-1.459595	-1.993233
H	2.543351	-2.082102	-2.877677
H	3.018596	-1.606369	-1.232798
C	1.154500	-3.042629	-0.465247
H	1.533956	-3.949061	-0.994161
H	0.177063	-3.278303	-0.035874
C	0.000000	-2.204922	-2.459251
H	0.215340	-3.182725	-2.967735
H	0.111711	-1.441880	-3.242186
C	-1.459852	-2.241011	-1.994072
H	-2.080940	-2.543698	-2.879436
H	-1.607148	-3.018711	-1.234000
C	-3.044836	-1.155194	-0.467450
H	-3.951067	-1.534464	-0.996913
H	-3.280872	-0.177945	-0.037785
C	-2.205544	0.000303	-2.460194
H	-3.183019	-0.214040	-2.969908
H	-1.441805	-0.110860	-3.242526
C	-2.241136	1.459595	-1.993233
H	-2.543351	2.082102	-2.877677
H	-3.018596	1.606369	-1.232798
C	-1.154500	3.042629	-0.465247
H	-1.533956	3.949061	-0.994161
H	-0.177063	3.278303	-0.035874
C	0.000000	2.204922	-2.459251
H	-0.215340	3.182725	-2.967735
H	-0.111711	1.441880	-3.242186
C	1.459852	2.241011	-1.994072
H	2.080940	2.543698	-2.879436
H	1.607148	3.018711	-1.234000

ScAAZTA

Sc	21.0	0.047224	-0.462986	-0.902760
O	8.0	-1.489354	-1.821518	-1.229360
O	8.0	-1.286551	0.931074	-1.724288
O	8.0	1.665971	0.534615	-1.767791
O	8.0	1.308571	-2.134082	-1.060998
O	8.0	3.222041	2.178798	-1.854350
O	8.0	-2.646111	2.650742	-1.179468
O	8.0	3.269951	-3.109296	-0.476124
O	8.0	-3.712692	-2.242809	-1.250520
N	7.0	0.441322	1.648448	0.316770
N	7.0	0.990409	-0.953093	1.283997
N	7.0	-1.613397	-0.392239	0.968480
C	6.0	0.033270	1.339525	1.721796
C	6.0	0.931846	0.180750	2.233744
C	6.0	0.103212	-2.056290	1.704779
H	1.0	0.452707	-2.497600	2.652935
H	1.0	0.152060	-2.821553	0.929554
C	6.0	-1.342548	-1.560188	1.841213
C	6.0	-1.446755	0.881001	1.692176
C	6.0	0.183853	2.531328	2.683853
H	1.0	1.201829	2.930326	2.648080
H	1.0	-0.024947	2.224594	3.714257
H	1.0	-0.507665	3.339775	2.433395
C	6.0	1.864536	1.991288	0.106234
C	6.0	2.307935	1.563863	-1.319519
C	6.0	-0.394663	2.668136	-0.372225
C	6.0	-1.568791	2.056495	-1.158102
C	6.0	2.344144	-1.466258	0.986914
C	6.0	2.334609	-2.339723	-0.301016
C	6.0	-2.898300	-0.572260	0.255421
C	6.0	-2.721803	-1.650530	-0.845867
H	1.0	0.554895	-0.146567	3.211909
H	1.0	1.942022	0.550603	2.418228
H	1.0	-1.576641	-1.299717	2.883514
H	1.0	-2.011814	-2.374963	1.560202
H	1.0	-1.809900	0.798918	2.729213
H	1.0	-2.057132	1.643504	1.205935
H	1.0	2.503542	1.451503	0.804885
H	1.0	2.063129	3.059484	0.250678
H	1.0	-0.762263	3.443064	0.307175
H	1.0	0.238351	3.163676	-1.112982
H	1.0	2.765686	-2.041897	1.822318
H	1.0	3.006483	-0.620109	0.786900
H	1.0	-3.711740	-0.865555	0.932386
H	1.0	-3.176545	0.360398	-0.238579

Scmacropa (DLD)

Si	0.257319	0.436598	0.087760
O	0.307481	-0.648802	-1.636431
O	-0.883234	0.607566	1.726571
O	2.199811	0.060558	1.688604
O	3.137995	-0.109092	-1.372474
O	1.281501	2.115676	-1.206134
O	0.958669	2.565343	1.644227
N	2.099654	-2.350304	-0.046230
N	-1.144481	2.748763	-0.246791
N	-0.520695	-1.816550	0.890591
N	-1.881053	0.322056	-0.915620
O	-0.625385	-2.281608	-2.890686
O	-2.863534	0.137862	2.679863
C	2.589882	-2.318711	1.324144
C	3.212857	-0.976953	1.646364
C	3.183129	-2.453420	-1.038540
C	3.127221	-1.349658	-2.079970
C	3.210175	1.028192	-2.207010
C	2.716068	2.192689	-1.379628
C	1.525277	2.052496	2.844919
C	2.680369	1.174063	2.460843
C	-0.703181	3.883542	0.592755
C	-0.161792	3.413571	1.922936
C	0.569362	3.083116	-1.999637
C	-0.923448	3.021838	-1.679431
C	1.045620	-3.322323	-0.288737
C	-2.549683	2.395622	0.062755
C	-2.922224	1.112933	-0.635474
C	-0.210922	-3.071759	0.516314
C	-2.026923	-0.732822	-1.728803
C	-3.268742	-1.140765	-2.196974
C	-4.382471	-0.383498	-1.822660
C	-4.207012	0.777566	-1.066040
C	-1.032243	-4.153067	0.864150
C	-2.188593	-3.939341	1.606965
C	-2.485010	-2.643312	2.026053
C	-1.617349	-1.625112	1.653491
H	1.376440	-4.357670	-0.086140
H	0.782916	-3.277102	-1.350696
H	-3.243643	3.197811	-0.232352
H	-2.633777	2.252154	1.143919
H	-3.333434	-2.003340	-2.849647
H	-5.374979	-0.665675	-2.159271
H	-5.045710	1.423923	-0.830676
H	-0.750457	-5.153470	0.552875
H	-2.833841	-4.770737	1.872388
H	-3.350497	-2.397125	2.629574
C	-0.697689	-1.315625	-2.146211
C	-1.854051	-0.200790	2.084185
H	3.336564	-3.106551	1.534542
H	3.681154	-1.036237	2.635785
H	3.970954	-0.689354	0.911264
H	-1.390449	3.964871	-2.005636
H	-1.376479	2.216338	-2.255291
H	0.726864	2.883065	-3.066432
H	0.984425	4.073104	-1.777502
H	-0.908107	2.850997	2.493156
H	0.167559	4.278391	2.515270
H	0.095982	4.418097	0.077628

H	-1.520237	4.602199	0.750939
H	4.006169	-1.421239	-2.738258
H	2.214542	-1.422817	-2.679529
H	3.163463	-3.426330	-1.548931
H	4.149673	-2.399623	-0.527102
H	3.426812	1.735967	1.882399
H	3.161201	0.806536	3.375307
H	0.771698	1.472554	3.391954
H	1.886719	2.872402	3.481964
H	1.756694	-2.475878	2.013278
H	2.974051	3.151512	-1.838781
H	3.157297	2.154577	-0.380401
H	4.242384	1.210576	-2.545901
H	2.572172	0.910898	-3.095181

Scmacropa (LDL)

Si	-0.547644	0.000000	0.000000
O	0.169703	0.408749	-1.880699
O	0.169703	-0.408749	1.880699
O	-1.736513	1.697631	1.427247
O	-2.514931	0.895254	-1.148432
O	-1.736513	-1.697630	-1.427247
O	-2.514931	-0.895253	1.148432
N	-0.512761	2.943251	-0.644528
N	-0.512762	-2.943251	0.644528
N	1.364734	1.426482	0.537410
N	1.364734	-1.426482	-0.537410
O	1.974221	0.839380	-3.162491
O	1.974221	-0.839381	3.162491
C	-0.868140	3.735199	0.545937
C	-1.002515	2.872446	1.800525
C	-1.499292	3.019989	-1.722276
C	-2.783557	2.297650	-1.346622
C	-3.330945	-0.030639	-1.874541
C	-2.431082	-1.065644	-2.506025
C	-3.330945	0.030640	1.874541
C	-2.431081	1.065645	2.506025
C	-1.499293	-3.019989	1.722276
C	-2.783558	-2.297648	1.346622
C	-1.002516	-2.872446	-1.800525
C	-0.868141	-3.735198	-0.545937
C	0.865256	3.156345	-1.088450
C	0.865255	-3.156345	1.088450
C	1.822455	-2.507320	0.113754
C	1.822456	2.507319	-0.113754
C	2.107049	-0.877363	-1.513550
C	3.387700	-1.316314	-1.826036
C	3.900728	-2.394781	-1.103768
C	3.100419	-3.012256	-0.143202
C	3.100420	3.012255	0.143202
C	3.900729	2.394780	1.103768
C	3.387701	1.316313	1.826036
C	2.107050	0.877362	1.513549
H	-1.820409	4.246697	0.372710
H	-0.125937	4.521320	0.735434
H	-1.556587	3.424284	2.569921
H	-0.035539	2.576802	2.214780
H	-1.074239	2.520695	-2.594876
H	-1.747713	4.059294	-1.999940
H	-3.515065	2.420001	-2.151524
H	-3.219693	2.689307	-0.423229
H	-3.901612	0.484628	-2.653525
H	-4.029500	-0.509658	-1.178252
H	-1.712836	-0.594018	-3.185502
H	-3.035523	-1.803706	-3.049474
H	-3.901612	-0.484627	2.653525
H	-4.029500	0.509659	1.178253
H	-3.035522	1.803708	3.049475
H	-1.712836	0.594018	3.185502
H	-1.074240	-2.520694	2.594876
H	-1.747714	-4.059294	1.999940
H	-3.219694	-2.689305	0.423229
H	-3.515066	-2.419999	2.151524
H	-0.035540	-2.576802	-2.214780
H	-1.556589	-3.424283	-2.569921

H	-1.820411	-4.246696	-0.372710
H	-0.125939	-4.521320	-0.735434
H	1.115348	4.225449	-1.184271
H	0.999539	2.702226	-2.073476
H	1.115346	-4.225449	1.184271
H	0.999538	-2.702226	2.073476
H	3.932386	-0.822596	-2.622032
H	4.897353	-2.772445	-1.309376
H	3.449975	-3.886138	0.396513
H	3.449976	3.886137	-0.396514
H	4.897354	2.772443	1.309376
H	3.932386	0.822595	2.622032
C	1.403036	0.218947	-2.277510
C	1.403036	-0.218947	2.277510

ScBispa[2]

Si	-1.381443	0.162372	-0.212572
O	-2.663914	1.558015	-0.947593
O	-3.163703	-0.585962	0.509103
N	-1.579671	1.690221	1.398949
N	-2.154394	-1.311804	-1.743893
N	-0.884713	-1.832942	1.440560
N	0.018732	2.393132	-0.756737
N	0.803338	0.326704	1.088801
N	0.479628	-0.631611	-1.731681
O	2.102368	-4.088580	0.032752
O	3.654331	-2.925352	1.192458
O	3.575214	2.123291	-2.393434
O	4.507147	1.774581	-0.363959
O	3.892679	-1.246108	-1.494068
H	4.596017	-1.807387	-1.142791
O	-4.268757	3.069539	-0.412168
O	-5.042566	-1.811349	0.202994
C	2.008168	-1.654379	0.027400
C	1.394627	-1.029150	1.320355
H	2.230304	-0.937176	2.029871
C	1.787143	1.232460	0.406250
H	2.592926	1.458393	1.117332
C	2.495093	0.590644	-0.840101
C	1.588233	0.308256	-2.045072
H	1.163140	1.231956	-2.426552
H	2.224875	-0.112851	-2.832107
C	1.032319	-1.886260	-1.147206
H	1.596238	-2.430036	-1.914963
H	0.208523	-2.525876	-0.832154
C	3.156868	-0.721417	-0.402766
H	3.814970	-0.531556	0.450668
C	2.569230	-3.036041	0.397112
C	4.232214	-4.181611	1.628506
H	5.088021	-3.910315	2.244158
H	4.541433	-4.774497	0.765650
H	3.498830	-4.749395	2.205132
C	3.574718	1.579236	-1.315330
C	5.572730	2.686509	-0.723550
H	6.229213	2.725025	0.144181
H	5.162230	3.673320	-0.948474
H	6.105564	2.313279	-1.600171
C	0.362410	-1.948230	1.927162
C	0.716412	-2.871281	2.910637
H	1.736953	-2.905599	3.277209
C	-0.259076	-3.738428	3.398742
H	-0.015391	-4.471477	4.160975
C	-1.553219	-3.628783	2.898320
H	-2.353425	-4.268288	3.253125
C	-1.826101	-2.658354	1.935814
H	-2.832178	-2.515892	1.569594
C	1.137967	2.528004	-0.025205
C	1.730078	3.763669	0.226642
H	2.628443	3.823594	0.831965
C	1.154042	4.906737	-0.330608
H	1.590807	5.884463	-0.154246
C	0.015927	4.766862	-1.120323
H	-0.459404	5.623839	-1.584037
C	-0.524311	3.492826	-1.298826
H	-1.431465	3.334433	-1.870411

C	0.449006	0.903114	2.436562
H	1.325639	1.391978	2.881795
H	0.188290	0.069531	3.093187
C	-0.727985	1.854509	2.411929
C	-0.949911	2.842605	3.371345
H	-0.252366	2.982928	4.190512
C	-2.083333	3.651651	3.245128
H	-2.282218	4.419842	3.985800
C	-2.929448	3.502027	2.143660
H	-3.786745	4.140469	1.965731
C	-2.623547	2.513541	1.215849
C	-3.303586	2.380049	-0.144924
C	-0.176180	-0.947430	-3.034653
H	0.499245	-1.525729	-3.680441
H	-0.364494	0.008520	-3.537627
C	-1.496502	-1.649783	-2.859551
C	-2.056816	-2.523141	-3.788942
H	-1.507304	-2.798899	-4.682564
C	-3.339308	-3.024595	-3.545472
H	-3.799250	-3.702231	-4.257802
C	-4.018420	-2.657301	-2.383190
H	-5.009578	-3.018939	-2.136357
C	-3.382352	-1.795495	-1.496995
C	-3.967149	-1.365312	-0.162502

ScL[2]

Sc	-1.335296	-0.115240	-0.371757
O	-3.264586	0.158116	-0.747469
N	-2.204270	1.074292	1.365666
N	-1.802746	-1.838597	-1.867238
N	-0.941350	-1.751347	1.198241
N	-0.807057	2.045484	-0.891452
N	0.475877	0.571800	1.112924
N	0.686710	-0.609917	-1.655398
O	2.616391	-3.517906	0.726131
O	4.055746	-1.974551	1.540907
O	3.118036	2.746634	-2.243760
O	3.895045	2.715364	-0.118113
O	4.094321	-0.384386	-0.980697
H	4.847160	-0.754537	-0.499880
O	-5.322235	0.967146	-0.245600
C	2.173723	-1.169424	0.312007
C	1.238150	-0.676522	1.454644
H	1.902535	-0.471112	2.305358
C	1.242997	1.636619	0.377108
H	1.855195	2.188693	1.098913
C	2.247593	1.094782	-0.691041
C	1.589894	0.535783	-1.955563
H	1.021798	1.309413	-2.468372
H	2.388769	0.216490	-2.633560
C	1.457380	-1.676114	-0.951596
H	2.230977	-2.065261	-1.624698
H	0.790404	-2.502265	-0.707462
C	3.113321	0.003390	-0.042458
H	3.572484	0.399567	0.869139
C	2.966820	-2.369007	0.864772
C	4.861681	-3.041365	2.122483
H	5.683615	-2.538321	2.627375
H	5.226457	-3.701281	1.333815
H	4.261153	-3.618441	2.827639
C	3.138160	2.275105	-1.132259
C	4.814652	3.795412	-0.443724
H	5.332234	4.024482	0.485538
H	4.260291	4.661172	-0.810741
H	5.514862	3.463021	-1.211673
C	0.227606	-1.729323	1.878599
C	0.495829	-2.634120	2.901478
H	1.440577	-2.586045	3.431606
C	-0.459463	-3.595689	3.229786
H	-0.267825	-4.311093	4.022639
C	-1.665970	-3.615205	2.530734
H	-2.440977	-4.337741	2.759027
C	-1.866465	-2.674972	1.527304
H	-2.793530	-2.647191	0.966815
C	0.265393	2.598573	-0.277159
C	0.507527	3.966833	-0.328528
H	1.375287	4.380984	0.173247
C	-0.370217	4.786763	-1.042184
H	-0.195976	5.856266	-1.098327
C	-1.470696	4.213159	-1.676691
H	-2.177172	4.813477	-2.238322
C	-1.660665	2.838778	-1.567906
H	-2.517948	2.344640	-2.013922
C	-0.040236	1.142483	2.410570

H	0.615967	1.949613	2.753931
H	0.013332	0.359352	3.172364
C	-1.471476	1.606877	2.347500
C	-2.034469	2.486614	3.273718
H	-1.425583	2.917771	4.061549
C	-3.392062	2.798010	3.166717
H	-3.851506	3.475434	3.879242
C	-4.146845	2.248193	2.126871
H	-5.197367	2.471035	1.978986
C	-3.502411	1.400865	1.235717
C	-4.149055	0.816122	0.001775
C	0.227887	-1.151842	-2.964793
H	1.070660	-1.585066	-3.518508
H	-0.143207	-0.306872	-3.557485
C	-0.892205	-2.144048	-2.814612
C	-1.046375	-3.232409	-3.668428
H	-0.287908	-3.451622	-4.412050
C	-2.196331	-4.014927	-3.560850
H	-2.341801	-4.868499	-4.214878
C	-3.164257	-3.668879	-2.617173
H	-4.088134	-4.228020	-2.524038
C	-2.931572	-2.572658	-1.795814
H	-3.672567	-2.230491	-1.082784

ScL3

Sc	1.200756	-0.811725	0.852057
O	3.129866	-1.023198	1.416878
O	0.722997	-2.017015	2.363536
N	2.532918	-0.614250	-1.040265
N	0.101391	-2.475922	-0.318304
N	1.672397	1.480940	0.653353
N	-0.120564	0.120561	-1.053575
N	-0.822729	0.069803	1.859059
O	5.356606	-1.119317	0.992487
O	-0.252925	-2.390496	4.370033
O	-4.172357	-2.004038	0.389353
O	-4.198526	-0.852225	-1.555732
O	-1.059131	4.190260	0.928753
O	-2.618223	3.763640	-0.649073
O	-3.827770	1.417988	0.662838
H	-4.207338	2.256257	0.366119
C	-2.416982	-0.374814	-0.044849
C	-1.363501	-0.708985	-1.141671
H	-1.863247	-0.516097	-2.099444
C	-0.312230	1.579512	-0.762083
H	-0.616603	2.086191	-1.689558
C	-1.454522	1.885072	0.273711
C	-1.121253	1.510023	1.727489
H	-0.270965	2.087834	2.083733
H	-1.988991	1.775175	2.345048
C	-1.994855	-0.722629	1.392927
H	-2.854254	-0.531646	2.045393
H	-1.758532	-1.782893	1.474092
C	-2.722603	1.126003	-0.168796
H	-2.950945	1.358528	-1.214859
C	-3.695777	-1.176792	-0.348239
C	-5.437879	-1.516448	-1.905219
H	-5.700562	-1.142431	-2.893304
H	-6.211050	-1.269847	-1.175252
H	-5.294878	-2.599202	-1.920828
C	-1.676812	3.405790	0.247003
C	-2.865092	5.190213	-0.757660
H	-3.642033	5.293373	-1.512910
H	-1.952289	5.705477	-1.062729
H	-3.195677	5.587224	0.203897
C	-0.958032	-2.174811	-1.093800
C	-1.684352	-3.161444	-1.756558
H	-2.529244	-2.884040	-2.377067
C	-1.312531	-4.496124	-1.592275
H	-1.864550	-5.283079	-2.095663
C	-0.230958	-4.804572	-0.768215
H	0.079809	-5.829458	-0.602176
C	0.449007	-3.763533	-0.143858
H	1.275721	-3.945311	0.534935
C	1.003576	2.176544	-0.291770
C	1.457212	3.408127	-0.751468
H	0.903292	3.938448	-1.518335
C	2.617425	3.953592	-0.199792
H	2.985845	4.915506	-0.540866
C	3.289459	3.247102	0.794362
H	4.191894	3.633477	1.253818
C	2.790403	2.006848	1.184314
H	3.298017	1.392278	1.919937
C	0.603691	-0.044531	-2.360270

H	0.237359	-0.960894	-2.831694
H	0.348199	0.775876	-3.042371
C	2.099229	-0.176584	-2.225242
C	2.987007	0.100373	-3.265308
H	2.618703	0.462789	-4.219552
C	4.353965	-0.092241	-3.049030
H	5.063154	0.109003	-3.845682
C	4.800045	-0.508033	-1.793885
H	5.848409	-0.629025	-1.547303
C	3.849191	-0.737466	-0.805667
C	4.196574	-1.005822	0.649110
C	-0.573108	-0.264539	3.290252
H	-1.480018	-0.154752	3.895561
H	0.182579	0.429316	3.677214
C	-0.018943	-1.697262	3.406852

ScEGTA

Sc	-0.003837	-0.120657	0.304862
O	-1.314660	-1.666735	0.946536
O	-0.255339	1.581970	1.428926
O	1.174953	-0.911722	1.831304
O	0.172377	1.266263	-1.344753
O	1.103840	-2.026074	-0.765374
O	-0.978100	-0.920936	-1.852777
N	2.369888	0.526163	-0.071434
N	-2.344352	0.607218	0.125732
O	-3.295898	-2.297192	1.835564
O	-1.037930	3.699787	1.433326
O	3.106790	-1.231990	2.960650
O	1.244422	2.855969	-2.534194
C	2.900816	-0.426602	-1.063039
H	3.998730	-0.340789	-1.145489
H	2.470892	-0.165230	-2.035053
C	2.531008	-1.877540	-0.758502
H	2.983012	-2.536386	-1.510660
H	2.880786	-2.192972	0.226235
C	0.542116	-2.652493	-1.918055
H	0.716371	-3.737088	-1.882177
H	0.998464	-2.240713	-2.829710
C	-0.946978	-2.337919	-1.921985
H	-1.402263	-2.703787	-2.854117
H	-1.440367	-2.779963	-1.050217
C	-2.136964	-0.179772	-2.232630
H	-2.676727	-0.697441	-3.038701
H	-1.738075	0.764384	-2.605398
C	-3.061445	0.049928	-1.041059
H	-3.898883	0.698232	-1.344628
H	-3.493340	-0.908616	-0.739981
C	2.336388	1.892264	-0.623027
H	3.283622	2.186707	-1.095706
H	2.126091	2.579318	0.201391
C	1.170739	2.041644	-1.618054
C	3.042287	0.426831	1.234369
H	2.875452	1.364574	1.770927
H	4.126697	0.273244	1.142918
C	2.417568	-0.683637	2.105513
C	-2.900213	0.034839	1.368694
H	-3.990777	0.151556	1.433436
H	-2.439302	0.544637	2.219258
C	-2.506006	-1.457186	1.415036
C	-2.242383	2.071527	0.159090
H	-3.180570	2.555034	0.467017
H	-1.973722	2.424338	-0.839321
C	-1.094919	2.525848	1.096005

ScBAPTA

Sc	0.000572	1.222905	0.000072
O	1.441945	2.637011	-0.102434
O	-1.440361	2.636983	0.101814
O	-0.495449	0.241518	1.682534
O	0.495850	0.241205	-1.682556
O	1.352937	-1.921792	0.318978
O	-1.352763	-1.920108	-0.317035
N	2.179660	0.419575	1.349383
N	-2.179793	0.419913	-1.349399
O	3.421384	3.599328	0.410598
O	-0.349455	-1.408410	3.209941
O	-3.420336	3.599218	-0.409421
O	0.349131	-1.407821	-3.210704
C	2.579817	-1.512534	-0.113974
C	3.030902	-0.283869	0.431419
C	4.252596	0.224512	-0.016100
H	4.595430	1.193754	0.323371
C	5.025561	-0.475295	-0.949454
H	5.965695	-0.048466	-1.285437
C	4.586986	-1.695786	-1.445993
H	5.178579	-2.242209	-2.174711
C	3.358249	-2.215390	-1.028084
H	2.991304	-3.144760	-1.445890
C	0.707406	-3.042007	-0.272174
H	1.216595	-3.975009	0.012839
H	0.694831	-2.944132	-1.363445
C	-0.708247	-3.041957	0.272047
H	-1.218232	-3.973981	-0.014673
H	-0.695405	-2.946078	1.363487
C	-2.580242	-1.511454	0.114721
C	-3.031359	-0.283160	-0.431524
C	-4.253404	0.225166	0.015070
H	-4.596128	1.194251	-0.324983
C	-5.026826	-0.474543	0.948106
H	-5.967330	-0.047896	1.283283
C	-4.588325	-1.694810	1.445301
H	-5.180337	-2.241115	2.173770
C	-3.359128	-2.214250	1.028516
H	-2.992150	-3.143264	1.447067
C	2.645017	1.757012	1.743218
H	3.659334	1.754418	2.161901
H	1.960993	2.114307	2.519712
C	2.542553	2.766864	0.581340
C	1.678869	-0.373853	2.498143
H	1.946938	0.127191	3.434981
H	2.135992	-1.361571	2.528828
C	0.150073	-0.559793	2.480520
C	-2.644474	1.757462	-1.743259
H	-3.658819	1.755484	-2.161888
H	-1.960253	2.114454	-2.519733
C	-2.541527	2.766954	-0.581139
C	-1.678989	-0.373749	-2.497864
H	-1.947502	0.126527	-3.435016
H	-2.135840	-1.361611	-2.527864
C	-0.150149	-0.559648	-2.480623

ScDTPA

Sc	-0.052322	0.099676	0.636396
N	2.420824	0.331339	-0.239943
N	0.103393	-0.992850	-1.631875
N	-2.407446	0.162633	-0.559214
O	0.184547	1.692301	-0.862795
O	1.240151	0.690127	2.143023
O	1.070120	-1.855394	0.746818
O	-1.407063	-1.366683	1.418313
O	-1.372265	1.625347	1.381410
O	1.332251	2.944107	-2.348257
O	3.220273	0.760584	3.227427
O	1.554176	-3.873289	-0.157584
O	-3.373636	-2.445682	1.709418
O	-2.727400	3.383519	0.940894
C	2.612829	-0.596040	-1.357170
H	2.835236	-1.583359	-0.955429
H	3.476167	-0.299424	-1.984373
C	1.393343	-0.671956	-2.290691
H	1.277956	0.288448	-2.794975
H	1.610045	-1.420811	-3.074119
C	-0.996991	-0.512400	-2.494634
H	-1.036598	-1.085281	-3.440932
H	-0.770271	0.528821	-2.732026
C	-2.367681	-0.571675	-1.823584
H	-3.124128	-0.203034	-2.541627
H	-2.629539	-1.613299	-1.612970
C	2.527892	1.736499	-0.636988
H	3.418820	1.935917	-1.255244
H	2.599326	2.337927	0.274942
C	1.253675	2.189761	-1.371090
C	3.214940	0.026200	0.952609
H	4.239250	0.427759	0.903703
H	3.259000	-1.058770	1.064915
C	2.523871	0.549830	2.234598
C	0.016337	-2.427148	-1.296646
H	0.223496	-3.066057	-2.170091
H	-0.989445	-2.643347	-0.929858
C	0.974488	-2.779289	-0.130153
C	-3.341983	-0.451916	0.394130
H	-4.242077	-0.851496	-0.097111
H	-3.670207	0.314317	1.101006
C	-2.668469	-1.544325	1.245027
C	-2.635776	1.607337	-0.660336
H	-3.688200	1.849855	-0.882159
H	-1.997302	2.023358	-1.440058
C	-2.219708	2.291445	0.673261

ScCHX-A″-DTPA

Si	-0.856694	0.711585	0.279609
O	-2.834935	1.345174	0.218216
O	-0.400528	0.231423	-1.821571
O	-1.589383	-0.684610	1.867619
O	-0.434585	2.655036	-0.512825
O	0.026357	1.330670	2.134696
N	-2.420661	-1.134718	-0.703878
N	0.452548	-1.462548	0.239353
N	1.723622	1.213534	0.038116
O	-5.074082	1.055221	0.302824
O	-0.674559	-0.891741	-3.759015
O	-1.205999	-2.449859	3.228631
O	0.649962	3.903230	-2.056338
O	1.638436	2.124020	3.508341
C	-1.837421	-2.440759	-0.394452
C	-0.306249	-2.483985	-0.531330
C	1.789685	-1.263053	-0.405073
C	2.533520	-0.018415	0.126121
C	3.915065	0.125413	-0.553188
C	4.786324	-1.125145	-0.410190
C	4.049395	-2.336805	-0.988202
C	2.694228	-2.513873	-0.295011
C	-3.704516	-0.871997	-0.047344
C	-3.921116	0.644674	0.168421
C	-2.463092	-0.853173	-2.140170
C	-1.061023	-0.487445	-2.655868
C	0.498133	-1.808653	1.676385
C	-0.893889	-1.645538	2.340615
C	1.742537	1.943200	-1.239600
C	0.551191	2.939439	-1.292437
C	2.034005	2.123232	1.153672
C	1.180211	1.830401	2.399410
H	-2.232502	-3.227387	-1.066961
H	-2.124240	-2.716273	0.618935
H	0.006039	-3.498891	-0.231962
H	-0.042294	-2.367174	-1.583722
H	1.563589	-1.070620	-1.458624
H	2.716341	-0.180151	1.196068
H	3.764020	0.327376	-1.620328
H	4.421331	1.003626	-0.136535
H	5.752900	-0.978450	-0.911671
H	5.004937	-1.306978	0.652356
H	4.651449	-3.249940	-0.882698
H	3.892613	-2.182798	-2.065401
H	2.870279	-2.735238	0.765849
H	2.174199	-3.381775	-0.713337
H	-3.672689	-1.309162	0.952371
H	-4.562426	-1.288647	-0.597643
H	-3.097546	0.026300	-2.290305
H	-2.882877	-1.689188	-2.723721
H	1.161482	-1.107076	2.187490
H	0.855955	-2.832745	1.853398
H	1.600018	1.243006	-2.062735
H	2.672344	2.509833	-1.395990
H	3.100713	2.119367	1.422072
H	1.780975	3.142202	0.851003

ScHOPO

Sc	0.070614	-1.551510	0.119145
O	-0.931655	-3.276473	1.168024
O	-1.805078	-0.895665	1.058557
O	-1.470765	-2.338902	-1.328469
O	-0.370834	-0.039191	-1.431394
O	1.053358	-1.661383	2.104798
O	0.516839	0.524564	0.852040
O	0.978469	-3.353444	-0.722269
O	2.250955	-1.198214	-0.453014
O	-5.816587	-0.038137	1.159034
O	6.222022	-0.827320	-1.527478
O	-1.049670	2.634500	-3.871019
O	0.381771	3.912932	2.194813
N	-2.709533	-1.865219	1.293218
N	-3.714247	0.811206	1.489162
N	-1.510468	-0.210318	-2.102353
N	-1.589238	2.964900	-1.669647
N	1.110052	0.612122	2.039688
N	1.970427	3.316249	0.662819
N	2.944294	-2.243902	-0.925861
N	4.564715	-0.019749	-0.185708
H	-2.734295	0.513289	1.471177
C	-2.164588	-3.155902	1.365441
C	-3.081019	-4.202510	1.657660
H	-2.663714	-5.199839	1.728632
C	-4.428176	-3.946458	1.809824
H	-5.114807	-4.763324	2.015643
C	-4.918532	-2.638742	1.691395
H	-5.967287	-2.389875	1.781914
C	-4.040751	-1.596640	1.438807
C	-4.605350	-0.195404	1.329848
C	-4.053644	2.189943	1.191495
H	-5.135916	2.295311	1.292532
H	-3.572023	2.836996	1.935499
C	-3.599223	2.592590	-0.218536
H	-3.891484	3.636128	-0.393679
H	-4.121871	1.977238	-0.959566
C	-2.081810	2.432799	-0.395843
H	-1.765106	1.394526	-0.306424
H	-1.556332	2.952284	0.411465
C	-2.080956	-1.488613	-2.029111
C	-3.286660	-1.686443	-2.746547
H	-3.729687	-2.673143	-2.685638
C	-3.862024	-0.654363	-3.463088
H	-4.791125	-0.818809	-4.001349
C	-3.256829	0.608976	-3.486740
H	-3.679371	1.440066	-4.036942
C	-2.085191	0.815978	-2.771384
C	-1.471618	2.203331	-2.799182
C	-1.464760	4.421674	-1.794288
H	-1.217455	4.626746	-2.837225
H	-2.446251	4.871000	-1.587713
C	-0.421593	5.031026	-0.857470
H	-0.621869	4.734239	0.175305
H	-0.536671	6.122939	-0.879464
C	1.020974	4.653095	-1.226600
H	1.030916	3.665075	-1.701010
H	1.411546	5.356928	-1.972515
C	1.952340	4.622231	-0.002350

H	1.651027	5.372206	0.733624
H	2.986081	4.838140	-0.297202
C	1.379466	-0.598752	2.698037
C	1.987168	-0.490735	3.974927
H	2.190933	-1.420245	4.492941
C	2.295891	0.743472	4.517328
H	2.755683	0.803013	5.499842
C	2.022512	1.918388	3.803690
H	2.254437	2.900799	4.195092
C	1.449712	1.823016	2.543583
C	1.184934	3.088818	1.750139
C	2.838626	2.300119	0.060765
H	2.297282	1.352986	0.038193
H	2.976747	2.576637	-0.989580
C	4.209665	2.204608	0.748791
H	4.075867	1.845645	1.775071
H	4.655109	3.204941	0.816376
C	5.170381	1.286228	-0.005176
H	6.119763	1.190501	0.536355
H	5.418692	1.709058	-0.989146
H	3.581017	-0.187366	0.045797
C	2.186105	-3.408734	-1.075823
C	2.847447	-4.538850	-1.619822
H	2.249547	-5.434327	-1.739268
C	4.181801	-4.477428	-1.969898
H	4.675204	-5.350745	-2.387260
C	4.902536	-3.292636	-1.778926
H	5.950570	-3.191381	-2.027776
C	4.274373	-2.177932	-1.244624
C	5.107014	-0.937662	-1.011982
